# A brief child-friendly reward task reliably activates the ventral striatum in two samples of socioeconomically diverse youth

**DOI:** 10.1371/journal.pone.0263368

**Published:** 2022-02-03

**Authors:** Melissa K. Peckins, Heidi B. Westerman, S. Alexandra Burt, Laura Murray, Martha Alves, Alison L. Miller, Ashley N. Gearhardt, Kelly L. Klump, Julie C. Lumeng, Luke W. Hyde

**Affiliations:** 1 Department of Psychology, St. John’s University, Queens, New York, United States of America; 2 Department of Psychology, University of Michigan, Ann Arbor, Michigan, United States of America; 3 Department of Psychology, Michigan State University, East Lansing, Michigan, United States of America; 4 McLean Imaging Center, McLean Hospital, Belmont, Massachusetts, United States of America; 5 Department of Psychiatry, Harvard Medical School, Boston, Massachusetts, United States of America; 6 Department of Family Medicine, Michigan Medicine, Ann Arbor, Michigan, United States of America; 7 Department of Health Behavior and Health Education, University of Michigan, Ann Arbor, Michigan, United States of America; 8 Department of Pediatrics, University of Michigan, Ann Arbor, Michigan, United States of America; 9 Institute for Social Research, University of Michigan, Ann Arbor, Michigan, United States of America; The University of Melbourne, AUSTRALIA

## Abstract

Adolescence is a period of increased risk-taking behavior, thought to be driven, in part, by heightened reward sensitivity. One challenge of studying reward processing in the field of developmental neuroscience is finding a task that activates reward circuitry, and is short, not too complex, and engaging for youth of a wide variety of ages and socioeconomic backgrounds. In the present study, we tested a brief child-friendly reward task for activating reward circuitry in two independent samples of youth ages 7–19 years old enriched for poverty (study 1: *n* = 464; study 2: *n* = 27). The reward task robustly activated the ventral striatum, with activation decreasing from early to mid-adolescence and increasing from mid- to late adolescence in response to reward. This response did not vary by gender, pubertal development, or income-to-needs ratio, making the task applicable for a wide variety of populations. Additionally, ventral striatum activation to the task did not differ between youth who did and did not expect to receive a prize at the end of the task, indicating that an outcome of points alone may be enough to engage reward circuitry. Thus, this reward task is effective for studying reward processing in youth from different socioeconomic backgrounds.

## Introduction

The transition from childhood to adolescence is characterized by an increase in risk-taking and reward-seeking behaviors that may be driven, in part, by heightened neural sensitivity to reward [1, 2]. The ventral striatum (VS), a key region activated during reward anticipation and receipt [3], reaches peak activation in response to winning versus losing during mid-adolescence [4]. Studies of reward processing that include children and adolescents can shed light on these normative developmental processes. However, one challenge of assessing reward processing in large-scale developmental studies that may be assessing multiple neural phenotypes is designing a task that reliably activates reward circuitry, produces individual differences in neural response to reward, is short, and is simple and engaging for children and adolescents.

The Monetary Incentive Delay task (MID) [5] is often used to study reward processing in adolescents and adults. During the MID feedback stage, the VS response to gains is similar for adolescents and adults [6]; however, the MID is complex and may not be engaging or fun for children. Moreover, to separate out anticipation and response phases requires relatively longer and more total trials, resulting in a relatively long task (i.e., >20 minutes) [7]. Researchers wanting to examine multiple neural circuits in the same study need brief, but engaging, tasks. One alternative to the MID is a simple and brief (<10 minutes) card guessing task, which uses a block design to increase power and combine reward and anticipation phases [8]. This task reliably activates reward circuitry in adults [8] and evokes individual differences in VS activity that predict outcomes such as problem drinking [9] and are predicted by experiences including early life stress [10]. However, this task was not designed to be engaging for youth and does not include gradations of reward magnitude, which may modulate the VS response [11]. Thus, the primary goal of this study was to create a child-friendly version of this task and examine whether it robustly engages reward circuitry in youth.

One key issue in validating child-friendly tasks relates to the samples used to examine these tasks. Human neuroscience has depended primarily on non-representative samples of convenience. Thus, even basic findings, such as regions activated by “standard” tasks, may only apply to primarily well-educated, European-American youth [12–14]. Moreover, understanding neural sensitivity to reward is key to understanding risk-taking, but studies have predominantly focused on relatively advantaged youth who are more sheltered from severe consequences of risk-taking. Risk-taking may present differently and have different consequences for youth living in disadvantaged contexts [15, 16]. Thus, we need tasks validated for youth living in these contexts [14]. This issue is particularly important given many neural reward tasks offer money as a prize, which may be differentially rewarding based on socioeconomic status [17]. Tasks that offer points as a prize, rather than money, may be less confounded by socioeconomic status. However, it is not clear whether points alone are a salient reward for youth or if a concrete prize is necessary to activate reward circuitry.

A second key issue is that gender and pubertal development may influence reward processing [18, 19]. For example, in one study, adolescent boys displayed stronger activation of reward circuitry when anticipating rewards, compared to girls [20]. Furthermore, adolescents in the early stages of puberty showed increased VS reactivity to reward compared to their more advanced peers [21]. At the same time, higher levels of testosterone have been associated with increased VS activation to reward in adolescent boys and girls [22] and greater estradiol was associated with lower caudate activation to reward cues in adolescent girls only [23]. Thus, when developing and validating neuroimaging tasks for children and adolescents, it is critical to examine whether gender and pubertal development modulate reward activation.

The primary goal of the present study was to validate a reward task that is appropriate for use in developmental studies and samples of youth with high rates of socioeconomic disadvantage. Our first aim was to test whether this brief, child-friendly card-flipping reward task would effectively activate the VS in two independent samples of youth 7 to 19 years old. Consistent with recent meta-analyses of reward processing [24, 25], we hypothesized that there would be greater VS activation to winning compared to losing. Given VS activation may differ according to reward magnitude [26], we expected VS activation would be greater when youth won larger versus smaller rewards and when youth lose small compared to large [27]. Since points themselves may not be rewarding enough, we also examined, experimentally, whether telling youth that they could win a concrete prize based on their point total would augment neural response to reward. Previous studies of youth suggest sociodemographic factors may influence reward processing. Our third aim was to test for effects of age, pubertal development, gender, pubertal development by gender interaction, and income-to-needs ratio on VS activation to reward [18, 19]. We expected older youth, youth with more advanced pubertal development [22], boys, and youth from households with a higher income-to-needs ratio would have greater VS activation to reward [28]. We tested our aims using a large, well-sampled study of twins enriched for neighborhood poverty and given the need for greater replication in psychology and neuroscience, replicated our findings in a younger sample of youth with high rates of socioeconomic disadvantage.

## Method

### Participants

#### Sample 1: Michigan twin neurogenetics study

Our first sample included a subsample of 354 twin pairs (708 youth; *n* = 386 boys, 322 girls; 220 DZ twin pairs) from the Michigan Twin Neurogenetics Study (MTwiNS) who previously participated in the Twin Study of Behavioral and Emotional Development in Children (TBED-C) within the Michigan State University Twin Registry [29–31]. Two cohorts of youth were recruited to participate in the TBED-C when they were 6–10 years old (*M* = 8.06 years, *SD* = 1.46). Youth in the first cohort were sampled from birth records to represent families living within 120 miles of Michigan State University. Youth in the second cohort were recruited from the same geographical area but only from neighborhoods with over 10.5% of families living below the poverty line (the mean at study onset). At the first assessment, youth in the first and second cohorts were ages 6 to 11.51 years old (*M* = 8.16 years, *SD* = 1.45) and 6 to 11.96 years old (*M* = 7.96 years, *SD* = 1.45), respectively. The MTwiNS sample was recruited from both samples, but only from those originally living in neighborhood with above average levels of poverty (i.e., the entire second sample, portions of the first) and thus represents families living in south-central Michigan with a substantial enrichment for neighborhood poverty. The MTwiNS sample was recruited when youth were 7 to 18 years old (*M* = 14.59 years, *SD* = 2.23).

The present study includes 464 youth (*n* = 263 boys, 201 girls) with a mean age of 14.63 years (*SD* = 2.14) who met fMRI eligibility criteria (S1 Table). Ages of youth ranged from 7 to 19 years old, though 97.84% of youth were between 10 and 18 years old and 95.69% of youth were between 12 and 17 years old (S1 Fig). Parents reported youths’ race as 76.72% White/Caucasian, 9.91% Black/African American, 9.05% Biracial, 2.16% Hispanic/Latino, 0.65% Asian/Pacific Islander, 0.43% Native American/Native Alaskan, and 1.08% other. Annual family household income ranged from $4,999 or less (0.43%) to $90,000 or more (38.79%), with a median annual income of $80,000 to $89,999 (12.26%). Nearly one-third of parents (32.11%) reported an annual household income of $50,000 to $59,999 or less, which falls below the living wage for a family of four (two working adults, two children) in Michigan ($66,393.60; Retrieved October 5, 2020, from http://livingwage.mit.edu/states/26) (Table 1).

**Table 1:** Demographic characteristics of the MTwiNS and ABC Brains samples.

	MTwiNS				ABC Brains			
Measure	*n*	*M* or *Median*	*SD*	Range	*n*	*M* or *Median*	*SD*	Range
Age (Months)	464	175.60	25.68	87–237	27	122.77	10.96	100–141
Age (years)	464	14.63	2.14	7.25–19.75	27	10.23	0.91	8.33–11.75
Pubertal Development^a^	464	2.96	0.80	1.17–4	27	1.73	0.57	1–3
Annual Household Income^b^	464	$80,000 to $89,999		$4,999 or less–$90,000 or more	27	$20,000 to $24,999		Under $5,000 –Greater than $75,000
Income-to-Needs Ratio^c^	464	2.40	0.92	0.09–3.72	27	1.18	0.92	0.08–3.05

^a^Pubertal development was measured via parent report with the Pubertal Development Scale [38]. Higher scores on the Pubertal Development Scale indicate later pubertal development, where 1 = “not yet started,” 2 = “barely started,” 3 = “definitely started,” and 4 = “seems complete”.

^b^Annual household income is parent report annual gross household income.

^c^Income-to-needs ratio was calculated by dividing the mid-point of each family’s income bracket by a poverty threshold based on interview year and number of people living in the household.

#### Sample 2: ABC brains study

Our second sample included a subsample of 56 youth from the ABC Brains study. Participants were originally recruited into the Appetite, Behavior, and Cortisol (ABC) Preschool cohort at ages 3–4 years from 2009–2011 from Head Start, a federally funded preschool program for low-income families [32]. Exclusion criteria for the original ABC Brains sample were: parent with ≥ 4-year college degree; parent/child not English-speaking; child in foster care, with food allergies, significant medical problems, or perinatal complications; gestational age < 35 weeks. The final, fMRI eligible, sample included 27 youth (*n* = 14 girls, 13 boys) ages 8 to 11 years old (*M* = 10.22 years, *SD* = 0.91; S1 Table; S1 Fig). Parents reported youths’ race as 62.96% White/Caucasian, 18.52% Hispanic, 7.41% Black/African American, and 11.11% other. Annual family household income ranged from less than $5,000 (7.41%) to greater than $75,000 (14.81%), with a median income of $20,000 to $24,999 (7.41%). The majority of parents (81.4%) reported an annual household income of $35,000 to $49,999 or less, which falls below the living wage for a family of four in Michigan ($66,393.60; Retrieved October 5, 2020, from http://livingwage.mit.edu/states/26) (Table 1).

### Procedure

The MTwiNS and ABC Brains study were approved by the Institutional Review Board of the University of Michigan. Similar procedures were followed for MTwiNS and ABC Brains. Youth and their primary caregivers visited the University of Michigan where, following written consent (primary caregiver) and assent (youth), they completed questionnaires and youth completed a one-hour MRI session which included the reward task. Prior to the MRI session, youth practiced the reward task in a mock scanner. To examine the effects of winning a prize based on reward task performance, a random subsample of MTwiNS youth were told before entering the scanner that they would receive a prize based on their point total after completing the reward task (*n* = 235, 50.65%) and the remainder learned about the prize after completing the task (*n* = 229, 49.35%). The prizes included $5.00 or a small toy (e.g., frisbee).

### Reward task

**Fig 1:**
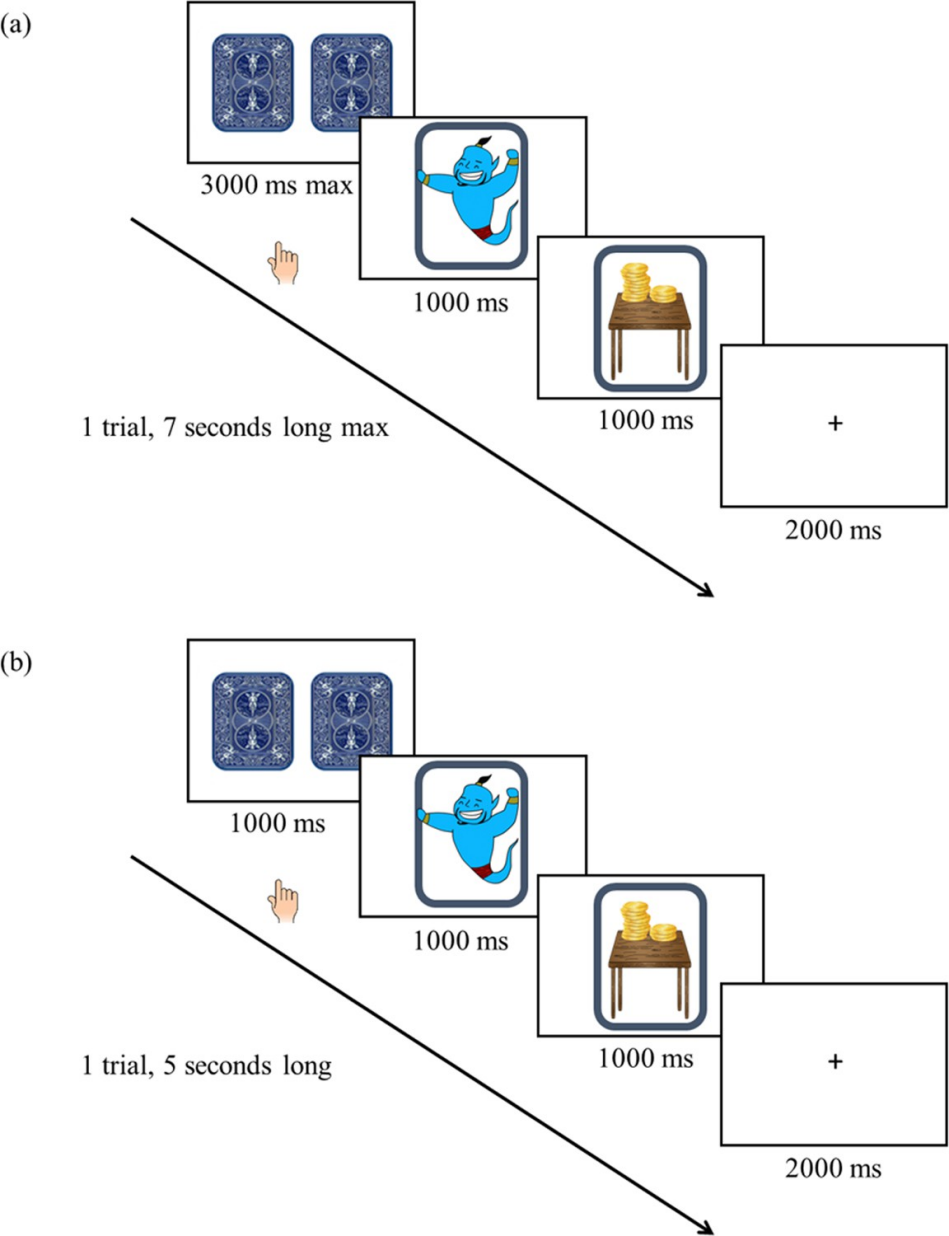
Child-friendly reward task. Participants were instructed to select one of two facedown cards to flip for a win, loss, or no change in points (neutral). The magnitude of the reward or loss could be large or small. Each block consisted of 4 identical and 1 incongruent trial. There were a total of 18 blocks; 3 blocks of large win, 3 blocks of small win, 3 blocks of large loss, 3 blocks of small loss, and 6 neutral blocks. Each set of 6 blocks occurred on a different island (turtle, parrot, monkey). Total run time of the task lasted approximately 10 minutes. **(a) Original version of the reward task for spiral acquisition.** The two facedown cards were displayed for up to 3000 ms. After the card flipped, one of three images appeared for 1000 ms: genie (win), pirate (loss), or palm tree (neutral). Feedback on the magnitude of the reward (large or small) was provided for 1000 ms. **(b) Modified version of the reward task for multiband acquisition.** The two facedown cards were displayed for 1000 ms. After the card flipped, an image of a genie (win), pirate (loss), or palm tree (neutral) was displayed for 1000 ms. Feedback on the magnitude of the reward (large or small) was provided for 1000 ms.

**Fig 2:**
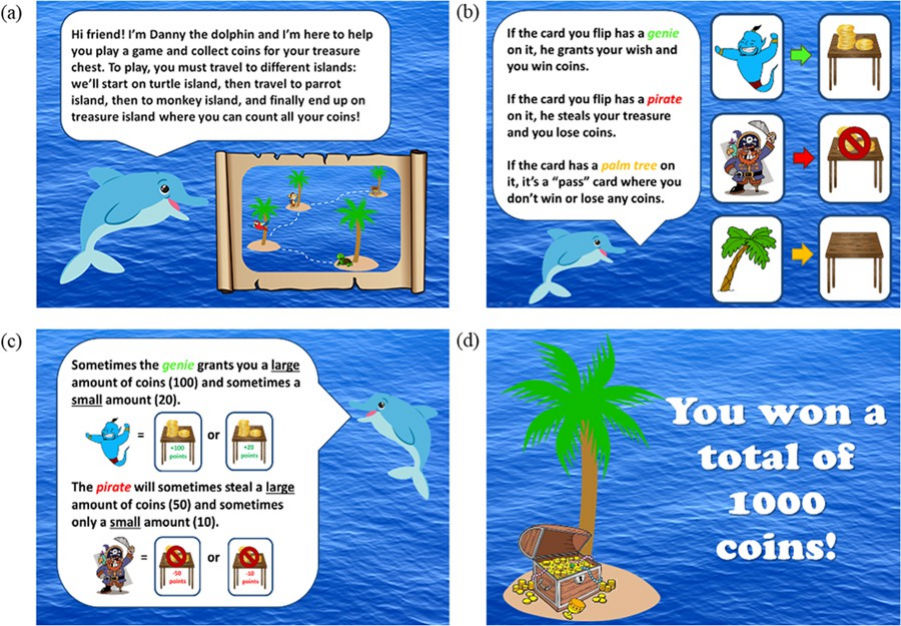
Selected images from the child-friendly reward task. Written and verbal instructions were provided to all participants. Participants progressed through the instruction screens at their own pace. **(a) Welcome screen explaining the task.** Participants are verbally instructed that they will be traveling to three different islands (turtle, parrot, monkey) to collect coins. **(b) Description of win, loss, and neutral trials.** Participants are told that they will see a genie if they win coins, a pirate if they lose coins, and a palm tree if they did not win or lose coins. **(c) Description of reward magnitude** Participants are instructed that they can win or lose large (i.e., +100 points or -50 points) or small (i.e., +20 points or -10 points). **(d) Final screen.** At the end of the task, participants travel to treasure island and are informed of their total winnings.

Youth completed a brief, child-friendly reward task that was a modified version of the paradigm developed by Hariri and colleagues [8] (Fig 1 and Fig 2). In this block-design task, youth played a fixed card game in which they selected one of two facedown cards to flip for a win, loss, or no change in points (neutral). The magnitude of the win or loss could be large (+100 points, -50 points) or small (+20 points, -10 points). The two facedown cards were displayed until youth selected a card to flip or 3000 ms passed, at which time a card automatically flipped. After flipping the card, one of three colorful cartoon images appeared for 1000 ms: a genie (win), pirate (loss), or palm tree (neutral). Feedback on the outcome magnitude was provided to youth for 1000 ms depending on if they won or lost a large or small number of points or the outcome was neutral. A fixation cross then appeared for 2000 ms. The maximum total trial length was 7000 ms, with youth selecting cards faster having shorter trials (Fig 1A). A slightly modified version of the reward task was played by some youth (*n* = 259, 55.82%) to reduce trial length variability. Two facedown cards were presented for 1000 ms and the total trial length was 5000 ms (Fig 1B). The modified version of the task can be downloaded from the Michigan Neurogenetics and Developmental Psychopathology (MiND) Laboratory website (https://sites.lsa.umich.edu/mindlab/research-projects/open-science/).

Each block consisted of 5 trials, 4 of which were the same type (e.g., large win block = 4 large win trials and 1 neutral trial), resulting in 90 trials. Each block contained one incongruent trial so youth would not know the outcome was fixed, yet still leverage the power of a block design. There were 18 blocks total: 3 large win, 3 small win, 3 large loss, 3 small loss, and 6 neutral. Each set of six blocks occurred on three different islands (turtle, parrot, monkey) to maintain child engagement. At the end of each island, a treasure chest with total coins earned was displayed. Total task run time was ~10 minutes. Participants with a response rate below 80% for MTwiNS (*n* = 54, 7.63%) and 90% for ABC Brains (*n* = 10, 17.86%) were excluded as this indicated a lack of task engagement (S1 Table). A different threshold was used for ABC Brains because task engagement was not monitored with an eye tracker.

### Imaging data acquisition

Blood oxygenated level dependent (BOLD) fMRI data were acquired using one of two research-dedicated GE Discovery MR750 3T scanners at the University of Michigan Functional MRI Laboratory. To leverage improvements in MRI data acquisition and to be consistent with the Adolescent Brain Cognitive Development (ABCD) study [33], we altered our acquisition protocol after the first 140 MTwiNS families (280 twins). For the first 140 MTwiNS families and all ABC Brains study participants, one run of 284 volumes was collected for each participant using an 8-channel head coil. BOLD functional images were acquired using a gradient-echo reverse spiral sequence (repetition time = 2000 ms, echo time = 30 ms, flip angle = 90°, FOV = 22 cm). Images included 43 interleaved oblique slices of 3mm thickness with 3.44 x 3.44mm^2^ in-plane resolution. High-resolution T1-weighted SPGR images (156 slices, slice thickness = 1 mm, in plane resolution of 1 x 1 mm^2^) were aligned with the AC-PC plane and used during normalization of the functional images. For the remaining MTwiNS participants (families 141–354), one run of 685 volumes was collected for each participant. BOLD functional images were acquired using a gradient-echo multiband sequence (repetition time = 800 ms, echo time = 30 ms, flip angle = 52°, FOV = 21.6 cm) with a 32-channel head coil, which covered 697 interleaved axial slices of 2.4 mm thickness. High-resolution T1-weighted SPGR images (298 slices, slice thickness = 1 mm) were aligned with the AC-PC plane and used during normalization of the functional images.

#### Preprocessing and quality control procedures

Preprocessing for both acquisition sequences were identical, unless otherwise specified. fMRI data were preprocessed and analyzed using SPM12 (Wellcome Department of Imaging Neuroscience, London, England). The first four volumes of each run were discarded to allow for stabilization of the MR signal. Raw k-space data were de-spiked before reconstruction to image space in reverse-spiral sequence acquisition. For gradient-echo sequence data with multiband acquisition, task-specific field maps were constructed from volumes of both anterior-to-posterior and posterior-to-anterior phase encoding; field maps were applied after image construction to reduce spatial distortions and minimize movement artifacts. Slice timing correction was performed using the 23^rd^ slice as the reference slice (reverse-spiral data) or the 2^nd^ slice of each 10-slice band (gradient-echo data with multiband acquisition). Functional data were then spatially realigned to the 10^th^ slice of the volume. These spatially realigned data were coregistered to a high-resolution T1-weighted image, segmented, and spatially normalized into standard stereotactic space to the Montreal Neurological Institute (MNI) template. fMRI data were smoothed using a 6mm Gaussian kernel.

After preprocessing, the Artifact Detection Tools toolbox (ART; https://www.nitr.org/projects/artifact_detect/) was used to detect translation or rotational motion outlier volumes that remained after earlier quality checks (> 2 mm movement or 3.5 rotation) and create regressors accounting for the possible effects of these volumes. Preprocessed images were also visually inspected for artifacts. Coverage of the VS was checked using a mask constructed by finding the intersection of a whole brain grey matter mask and a large bilateral VS mask. The whole brain grey matter mask was created using FSL’s FAST [34] to segment the scalped template brain in SPM12. The bilateral VS mask was constructed with the Talairach Daemon option of the WFU PickAtlas Tool version 2.4 (RRID: SCR_007378) [35]. Two 10-mm radius spheres were created around MNI coordinates *x* = ±12, *y* = 12, *z* = -10 to encompass the right and left VS [9]. AFNI’s 3dcalc tool [36] was used to identify the common areas between the whole brain grey matter mask and bilateral VS mask to create a grey matter VS mask. A participant’s fMRI data was considered unusable if VS coverage was less than 70% for MTwiNS and less than 90% for ABC Brains (S1 Table). The MTwiNS threshold was lower due to increased susceptibility to artifacts associated with multiband echo-planar imaging acquisition. No MTwiNS participants with spiral sequencing had less than 90% VS coverage. In MTwiNS and ABC Brains, included and excluded participants did not differ on gender, age, pubertal development, and household income (*p* > .05).

#### Imaging data analysis

In the MTwiNS sample, we checked for confounding by scanner sequence (i.e., gradient-echo reverse spiral sequence vs. gradient-echo-planar sequence with multiband acquisition) during total win vs. total loss trials, our most powerful contrast. When scanner sequence was entered as a predictor variable, it did not predict activity in the VS, nor in any clusters in reward processing centers across the whole brain at *p* < .001 with and without controlling for age and gender. Since there were no confounding effects, scanner type was not included as a covariate in the analyses.

fMRI data were modeled using the general linear model in SPM12. The following conditions were modeled: total win trials, total loss trials, neutral trials, large win trials, small win trials, large loss trials, small loss trials. Though our primary contrast of interest was total win vs. total loss to maximize VS response [8], we also modeled total win vs. neutral, neutral vs. total loss, large win vs. small win, and small loss vs. large loss to examine whether responses were modulated by win versus loss and reward magnitude [26, 37]. We focused on VS activation via a region of interest (ROI) approach but report whole brain analyses to best characterize the task and promote open science. For the exploratory whole brain analyses, participants with less than 90% coverage in a grey matter PFC mask and/or the whole brain grey matter mask were excluded, resulting in a final whole-brain subsample of 446 for MTwiNS (*n* = 18 excluded, 3.88%) and 21 for ABC Brains (*n* = 6 excluded, 22.22%). The PFC mask was created with AFNI’s 3dcalc tool [36] to find the common areas between the whole brain grey matter mask and a frontal lobe mask generated by WFU PickAtlas (RRID: SCR_007378) [35]. Results were visualized using SPM12 and the xjView toolbox (https://www.alivelearn.net/xjview).

### Measures

#### Demographic characteristics

Youth *gender* and *age* (months) were reported by the primary caregiver. *Pubertal development* was assessed via primary caregiver report with the Pubertal Development Scale [38]. Parents reported on youths’ change in growth, body hair, and skin. Parents also reported on boys’ change in voice and facial hair, and girls’ breast development and menarche status. All items except menarche status were on a 4-point scale ranging from “has not yet begun” to “seems completed.” Menarche status was recoded so that 1 represents “has not started” and 4 represents “has started” menarche. Pubertal development scores were created by calculating the mean of boys’ change in growth, body hair, skin, voice, and facial hair, and the mean of girls’ change in growth, body hair, skin, breast development, and menarche status. Mean pubertal development scores ranged from 1 to 4, with higher values reflecting later stages of development. *Income-to-needs ratio* was calculated by dividing the mid-point of each family’s annual household income bracket by the poverty threshold. Primary caregivers reported their family’s annual household income on a 13-point scale ranging from “$4,999 or less” to “$90,000 or more” for MTwiNS and a 9-point scale ranging from “under $5,000” to “greater than $75,000” for ABC Brains. Poverty thresholds were assigned to each family based on interview year and number of people living in the family’s household (https://aspe.hhs.gov/).

### Analytic plan

We used an ROI approach using an anatomical bilateral, grey matter VS mask [9]. First, we tested whether the VS was activated in response to the reward task and if the VS response to win or loss was modulated by reward magnitude without including covariates in the models. Individual contrast files were used in a second-level random effects model to determine group mean VS activation using a one-sample t-test. Next, we tested whether mean VS activation differed between the two prize conditions using a two-sample t-test in the MTwiNS sample. Then, in the MTwiNS sample, for the contrasts comparing total win, total loss, and neutral, we tested whether VS activation differed according to gender, linear and quadratic age, age by gender interaction, linear and quadratic pubertal development (controlling for gender), pubertal development by gender interaction, and income-to-needs ratio using eight separate multiple regression models for each contrast. In the ABC Brains sample, for the total win vs. total loss contrast only, we tested whether VS activation differed according to gender, age, pubertal development (controlling for gender), and income-to-needs ratio using separate multiple regression models. To limit the number of tests performed, we only tested for interactions in the ABC Brains sample that were significant in the larger MTwiNS sample.

For full transparency and to promote replicability, in the larger MTwiNS sample, we tested for main effects of the reward task across the whole brain and whether the neural response to win or loss was modulated by reward magnitude. In the ABC Brains replication sample, we only tested for main effects of the reward task across the whole brain for the total win vs. total loss contrast, the most powerful contrast. Finally, in the MTwiNS sample, we confirmed that our results were not due to ages at the extreme ends of the distribution by testing for main effects of task and associations between demographic characteristics and activation in the VS with youth between the ages of 10 and 18 years old (*n* = 454). We also confirmed our findings were not impacted by the nesting of twins within families by testing for main effects of task using 10 randomly generated subsamples of single twins. The genetically independent subsamples included all twins who did not have a co-twin scanned (*n* = 90) and a randomly selected twin from all complete twin pairs (*n* = 187), resulting in a final subsample of 277 youth. We are submitting contrast maps of second-level analyses to Neurovault (https://neurovault.org).

We used a 3dClustSim correction within SPM12 to achieve a statistical threshold of *p* < .05 small volume corrected for multiple comparisons using a voxel level *p* < .001 for MTwiNS and *p* < .01 for ABC Brains, using an updated version of 3dClustSim [39] in AFNI version 16.1.14 [36]. A lower voxel significance threshold was used for ABC Brains because of the small sample size (*n* = 27). We implemented the spatial autocorrelation function to model the spatial smoothness of noise volumes. Group-level smoothing values were estimated from a random 10% of participants’ individual-model residuals, using the program 3dFWHMX. 3dClustSim uses a Monte Carlo simulation to provide thresholds that achieve a family-wise error of *p* < .05 within the VS and whole brain grey matter mask.

## Results

### MTwiNS main effects

**Fig 3:**
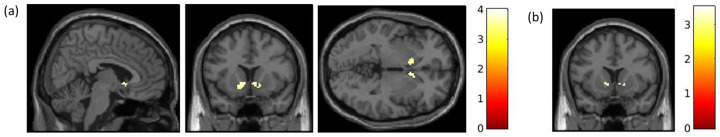
The ventral striatum is robustly activated during a child-friendly reward task in the MTwiNS sample (*n* = 464). k = number of voxels within the cluster. Significant clusters were identified in SPM12 using a mask of the ventral striatum [9], grey matter segmented using AFNI [36]. False positive rate is controlled across the ventral striatum mask using 3dClustSim for cluster-level correction (*p*_unc_ < .001, alpha < .05, k > 3). **(a) Ventral striatum activation during total win > total loss trials.** Left: k = 77, T = 3.83, MNI -14, 10, -8. Right: k = 72, T = 4.02, MNI 6, 12, -2. **(b) Ventral striatum activation during total win > neutral trials.** Left: k = 12, T = 3.52, MNI -8, 12, -2. Right 1: k = 6, T = 3.34, MNI 14, 10, -2. Right 2: k = 3, T = 3.32, MNI 10, 12, -2.

The reward task robustly activated the VS in the MTwiNS sample of youth (Table 2). As hypothesized, the task elicited greater bilateral VS activation during win compared to loss and neutral trials (Fig 3; 3dClustSim *p*_unc_ < .001, alpha < .05). Excluding participants younger than 10 (*n* = 9) and older than 18 (*n* = 1) did not substantially change the main effects of task (S2 Table). VS activation did not differ according to reward magnitude.

**Table 2:** Main effects of task in the ventral striatum.

Study	*N*	Contrast	Peak (x, y, z)	T	k
MTwiNS	464	Total Win > Total Loss	6, 12, -2	4.02	72
			-14, 10, -8	3.83	77
		Total Win > Neutral	-8, 12, -2	3.52	12
			14, 10, -1	3.34	6
			10, 12, -2	3.32	3
ABC Brains	27	Total Win > Total Loss	8, 14, -2	3.27	108
		Large Loss > Small Loss	-16, 12, -6	2.85	56

k = number of voxels within the cluster. Significant clusters were identified in SPM12 using a mask of the ventral striatum [9], grey matter segmented using AFNI [36]. False positive rate is controlled across the ventral striatum using 3dClustSim for cluster-level correction (MTwiNS *p*_unc_ < .001, alpha < .05, k > 3; ABC Brains *p*_unc_ < .01, alpha < .05, k > 32).

To test for general reliability of task-evoked activation within twin pairs, we extracted activation from significant clusters within the VS during win compared to neutral and loss trials and tested for correlations between all complete twin pairs (*n* = 187 pairs), monozygotic twin pairs (*n* = 77 pairs), and dizygotic twin pairs (*n* = 110 pairs; S3 Table). Extracted right VS activation during win compared to neutral trials was positively, albeit weakly, correlated for all complete twin pairs (*r* = .110, *p* = .040) and trending for monozygotic twin pairs, (*r* = .144, *p* = .075), and in the same direction, though not significant for dizygotic twin pairs (*r* = .078, *p* = .251). Extracted left VS activation during win compared to neutral trials was not correlated for all complete twin pairs (*r* = .010, *p* = .840) or dizygotic twin pairs (*r* = -.064, *p* = .348), but was trending in the positive direction for monozygotic twin pairs (*r* = .160, *p* = .051). Interestingly, during win compared to loss trials, only extracted right VS activation was correlated at a trending level for monozygotic twin pairs (*r* = .087, *p* = .090). All other correlations with extracted right and left VS activation during win compared to loss trials were not significant (*p*s = .140 to .690).

**Fig 4:**
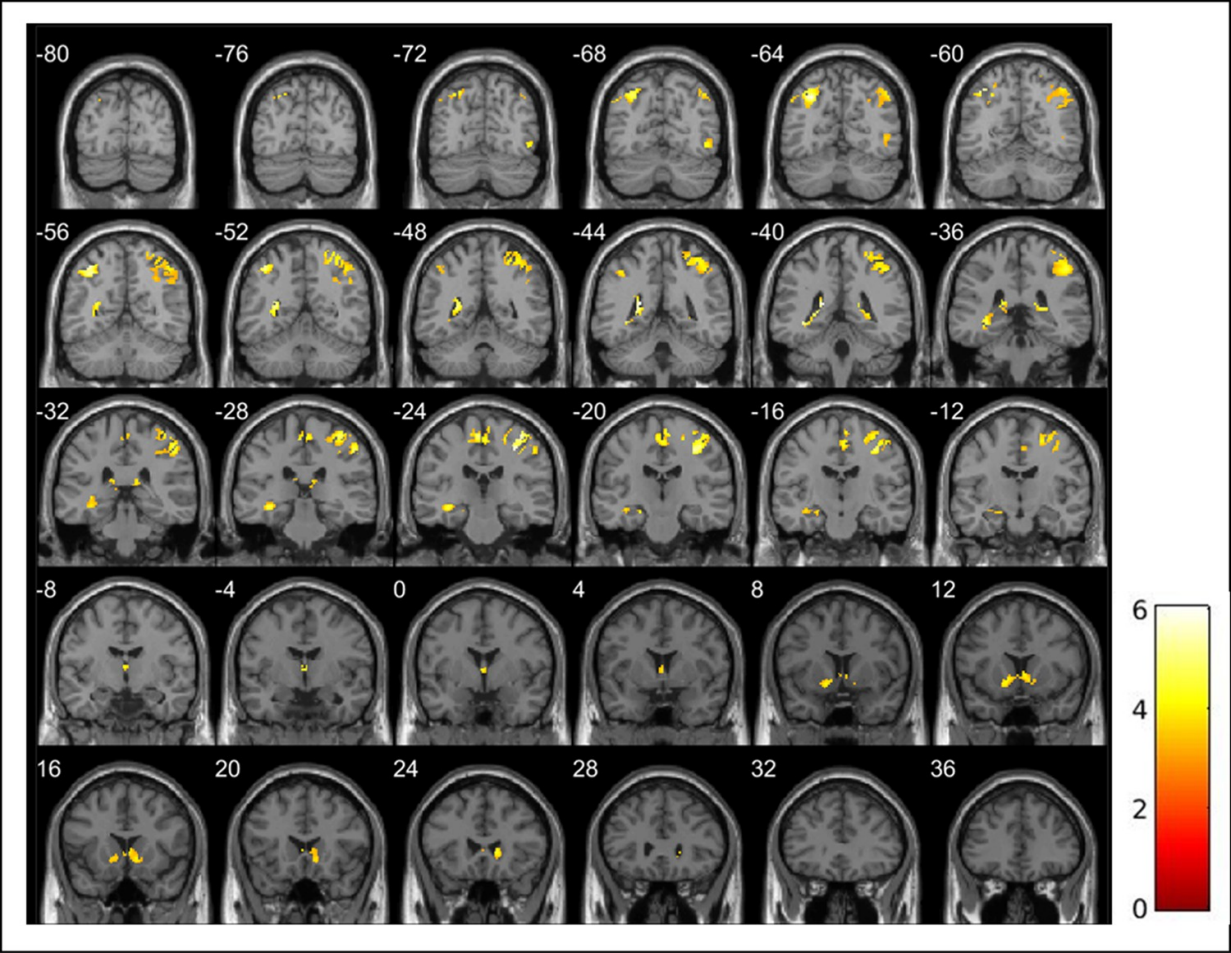
Whole brain activation during total win > total loss trials during a child-friendly reward task in the MTwiNS sample (*n* = 446). k = number of voxels within the cluster. False positive rate is controlled using 3dClustSim for cluster-level correction (*p*_unc_ < .001, alpha < .05, k > 57).

**Fig 5:**
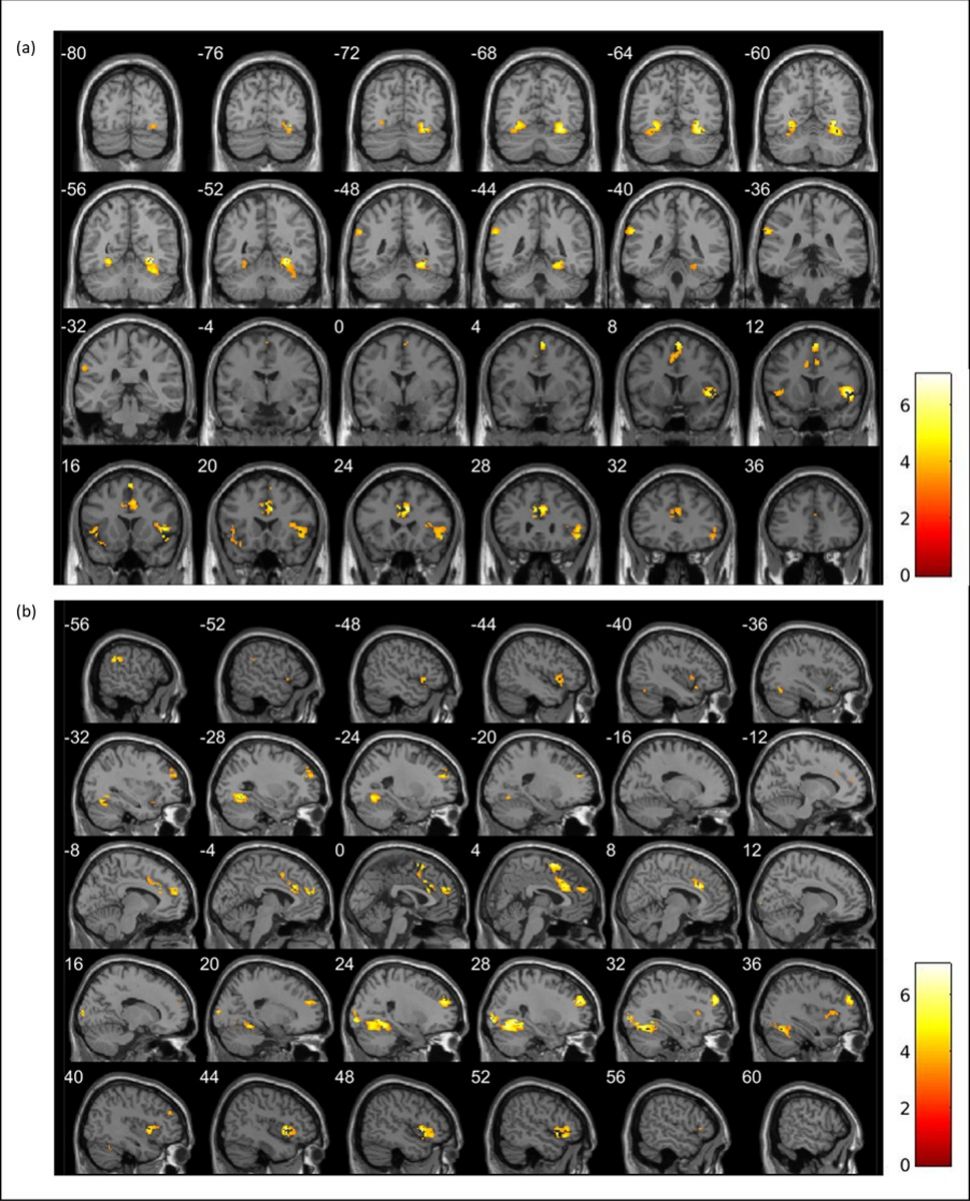
Whole brain activation during total loss > total win trials during a child-friendly reward task in the MTwiNS sample (*n* = 446) shown in (a) coronal view and (b) sagittal view. k = number of voxels within the cluster. False positive rate is controlled using 3dClustSim for cluster-level correction (*p*_unc_ < .001, alpha < .05, k > 57).

Whole brain analyses revealed engagement of core areas for reward processing including the caudate, putamen, nucleus accumbens, thalamus, and supplemental motor area during win compared to loss trials (3dClustSim *p*_unc_ < .001, alpha < .05; Table 3; Fig 4) [24]. There was overlap between the significant clusters of activation across the whole brain during win compared to loss trials and the bilateral VS mask used for ROI analyses (S2 Fig). During loss compared to win trials, greater activation was found in regions associated with loss anticipation including the insula and supplemental motor area (Table 3, Fig 5) [24]. Whole brain analyses for neutral compared to win and loss trials revealed robust activation across the brain (S4 Table). Specifically, during win compared to neutral trials, we found greater activation in the insula, putamen, supplemental motor area, and thalamus and 4 voxels overlapped with the left VS mask used for ROI analyses (S2 and S3 Figs). During neutral compared to loss trials, activation was greater in the caudate, insula, supplemental motor area, and thalamus (S4 and S5 Figs). Activation across the brain was also modulated by reward magnitude (S5 Table). Activation was greater in the insula and supplemental motor area during large win compared to small win trials and left lingual gyrus during large loss compared to small loss trials (S6 and S7 Figs).

**Table 3:** MTwiNS whole brain main effects of task for total win compared to total loss.

Contrast	Side	Region Labels	Peak (x,y,z)	T	k
Total Win > Total Loss	Left	Precuneus, Hippocampal gyrus, Hippocampus	-22, -44, 14	6.04	514
	Left	Inferior parietal gyrus, Superior parietal gyrus	-36, -56, 52	5.91	464
	Right	Postcentral gyrus, Precentral gyrus, Inferior parietal gyrus, Superior parietal gyrus	32, -24, 48	5.86	1679
	Left	Paracentral lobule, Supplementary motor area	-4, -22, 60	5.09	279
	Right	Paracentral lobule, Supplementary motor area			
	Left	Thalamus, Caudate, Putamen, Nucleus accumbens	0, -6, 8	5.03	362
	Right	Thalamus, Caudate, Putamen, Nucleus accumbens			
	Right	Hippocampus, Precuneus	22, -38, 10	4.95	93
	Right	Inferior temporal gyrus, Inferior occipital gyrus	46, -70, -6	4.51	88
	Right	Precentral gyrus	32, -20, 64	4.16	134
Total Loss > Total Win	Right	Lingual gyrus, Fusiform gyrus	28, -54, -6	7.08	1079
	Right	Middle cingulate and paracingulate gyri, Anterior cingulate cortex, Supplementary motor area	8, 24, 30	6.45	606
	Left	Middle cingulate and paracingulate gyri, Anterior cingulate cortex, Supplementary motor area			
	Right	Insula, Inferior frontal gyrus pars orbitalis, Inferior frontal gyrus (triangular part)	48, 12, 0	6.29	596
	Right	Middle frontal gyrus, Superior frontal gyrus (dorsolateral)	30, 50, 26	5.93	440
	Left	Fusiform gyrus, Lingual gyrus	-28, -58, -8	5.40	340
	Left	Superior frontal gyrus (medial), Anterior cingulate cortex	0, 50, 26	4.96	254
	Right	Superior frontal gyrus (medial)			
	Left	Middle frontal gyrus, Superior frontal gyrus (dorsolateral)	-22, 48, 26	4.92	194
	Left	SupraMarginal gyrus	-58, -44, 30	4.85	198
	Left	Inferior frontal gyrus (triangular part), Insula	-46, 16, 0	4.33	142

*n* = 446. k = number of voxels within the cluster. False positive rate is controlled for using 3dClustSim for cluster-level correction (*p*_unc_ < .001, alpha < .05, k > 57). Anatomical region labels were retrieved from the AAL3 atlas [40]. The anatomical regions listed are not exhaustive, but full activation can be seen in Figs 4 and 5.

#### Prize condition

VS activation did not differ between youth who learned about the prize before (*n* = 235) and after completing the task (*n* = 229). Whole brain analyses revealed that compared to youth who learned about the prize after completing the task (*n* = 220), youth who knew about the prize before the task (*n* = 226) had greater activation in the occipital lobe during large win compared to small win trials (S8 Fig).

#### Demographic characteristics

**Fig 6:**
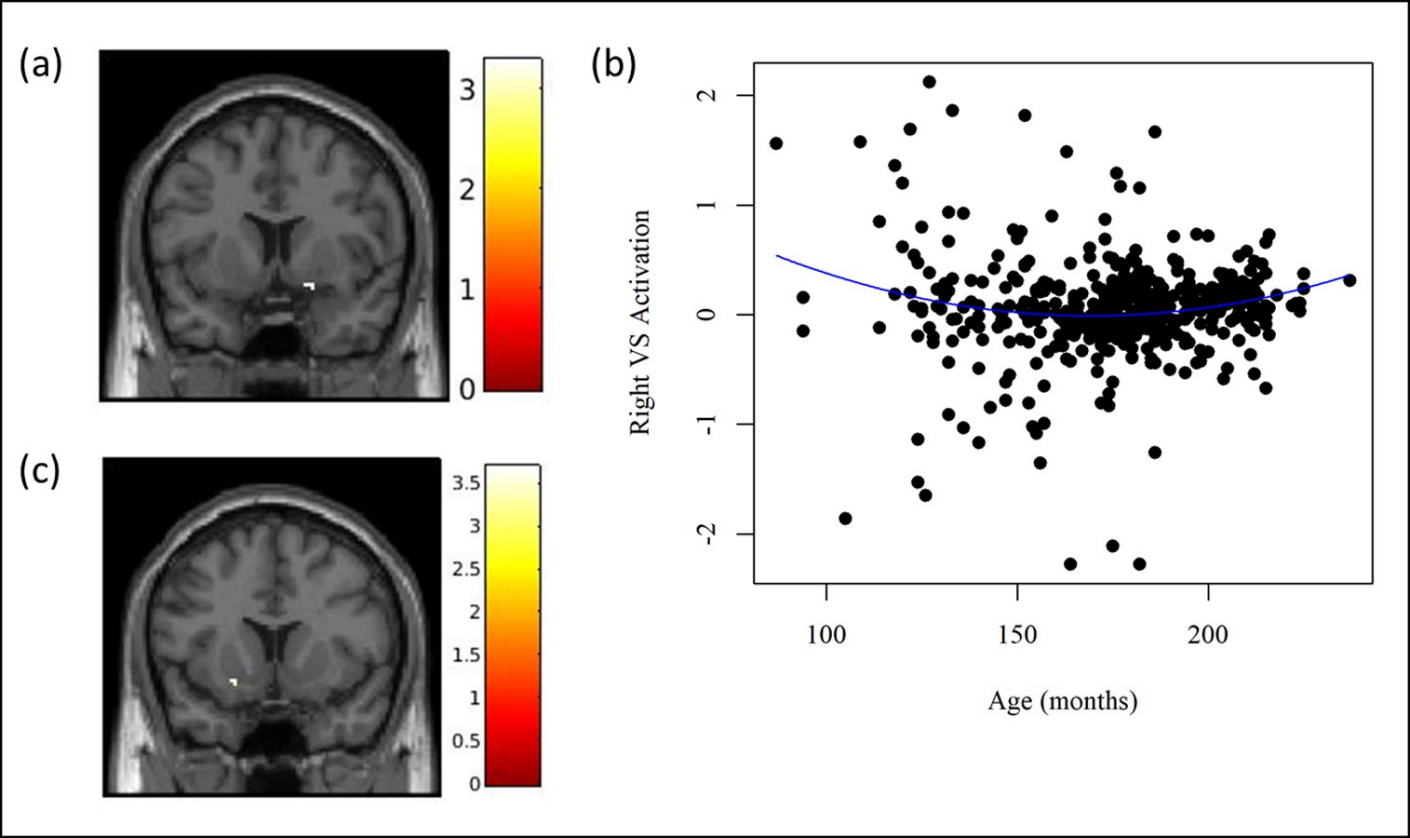
Relationship between age and ventral striatum activation during total win > neutral trials in the MTwiNS sample. k = number of voxels within the cluster. Significant clusters were identified in SPM12 using a mask of the ventral striatum [9], grey matter segmented using AFNI [36]. False positive rate is controlled across the ventral striatum using 3dClustSim for cluster-level correction (*p*_unc_ < .001, alpha < .05, k > 3). **(a) Quadratic association between age and ventral striatum activation during total win > neutral trials (*n* = 464).** Right: k = 7, T = 3.29, MNI 16, 8, -12. **(b) Plot of the quadratic age effect with extracted ventral striatum activation (*n* = 464).** VS = ventral striatum. To interpret the quadratic age effect, right VS activation was extracted from a mask of the significant cluster during total win > neutral trials and plotted by age in months with a quadratic curve. The association between age and VS activation followed a “U” shaped curve, with activation decreasing from early- to mid-adolescence and increasing from mid- to late adolescence. **(c) Linear association between age and ventral striatum activation during total win > neutral trials in a subsample of MTwiNS youth ages 10 to 18 years old (*n* = 454).** MTwiNS youth were excluded from analyses if they were younger than 10 (*n* = 9) or older than 18 years old (*n* = 1) to confirm that our results were not due to ages at the extreme ends of the distribution. Left: k = 6, T = 3.70, MNI -22, 12, -10.

In eight separate regression models, we tested whether VS activation to the reward task was associated with age, gender, age by gender interaction, pubertal development, pubertal development by gender interaction, and income-to-needs ratio. There were no significant clusters in the VS above *p*_unc_ < .001 associated with gender, the age by gender interaction, pubertal development, the pubertal development by gender interaction, and income-to-needs ratio. There was a quadratic relationship between age and VS activation during total win compared to neutral trials, with activation decreasing from early to mid-adolescence and increasing from mid- to late adolescence (Fig 6A and 6B). In the subsample of youth restricted to 10 to 18 years old (*n* = 454), there was no quadratic relationship between age and VS activation during total win compared to neutral trials, but there was a linear association, though on the opposite side (i.e., right vs. left) of the brain (S2 Table; Fig 6C).

#### Subsample of genetically independent twins

We tested for differences in VS activation between win compared to loss and neutral trials in 10 randomly generated subsamples of genetically independent twins from the MTwiNS sample (*n* = 277) to confirm that our results were not impacted by the nesting of twins within families (S6 Table). Of the 10 randomly selected draws, findings held up in six subsamples for win compared to loss trials (3dClustSim *p*_unc_ < .001, alpha < .05). That is, we found greater activation in the VS during win compared to loss trials. For win compared to neutral trials, results held up in four subsamples (3dClustSim *p*_unc_ < .001, alpha < .05), with one more subsample trending in the left VS (3dClustSim *p*_unc_ < .001, alpha < .10). Specifically, VS activation was greater during win compared to neutral trials. Thus, the overall pattern of findings in these subsamples was similar, albeit with lower statistical significance due to the lower sample size and power.

### ABC Brains main effects

**Fig 7:**
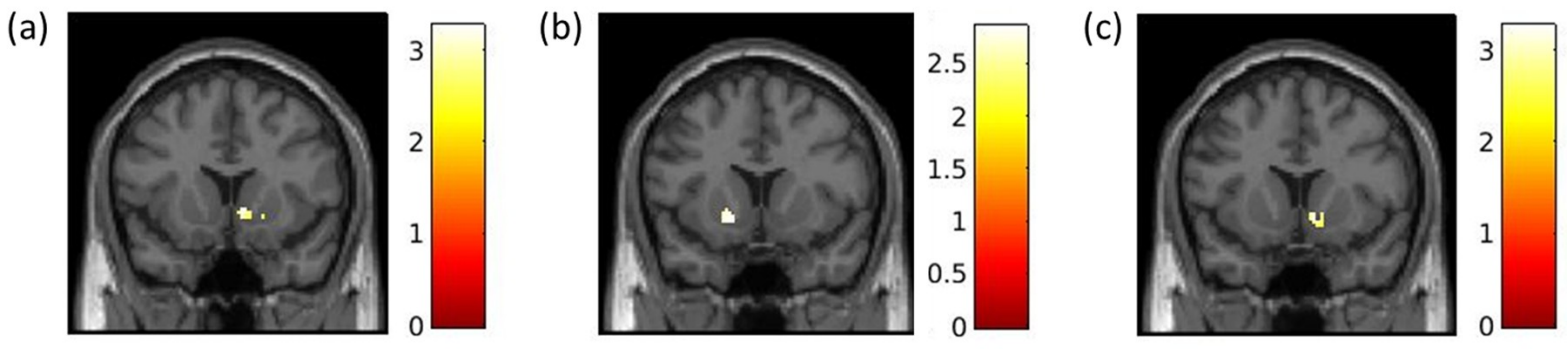
The ventral striatum is robustly activated during a child-friendly reward task in the ABC brains sample (*n* = 27). k = number of voxels within the cluster. Significant clusters were identified in SPM12 using a mask of the ventral striatum [9], grey matter segmented using AFNI [36]. False positive rate is controlled across the ventral striatum mask using 3dClustSim for cluster-level correction (*p*_unc_ < .01, alpha < .05, k > 32). **(a) Ventral striatum activation during total wins > total losses trials.** Right: k = 108, T = 3.27, MNI 8, 14, -2. **(b) Ventral striatum activation during large loss > small loss.** Left: k = 56, T = 2.85, MNI -16, 12, -6. **(c) Pubertal development was positively associated with ventral striatum activation during total wins > total losses trials when controlling for gender.** Right: k = 42, T = 3.34, MNI 10, 12, -6.

The reward task activated the VS in the ABC Brains sample, replicating the MTwiNS finding, albeit at a lower statistical threshold and only for the most powerful contrast (Table 2). Right VS activation was greater during win compared to loss trials (3dClustSim *p*_unc_ < .01, alpha < .05; Fig 7A). Left VS activation was greater during large loss compared to small loss trials (Table 2; Fig 7B). VS activation did not differ when comparing neutral trials with win and loss trials and large win with small win trials. In the ABC Brains replication sample, we only tested for main effects of the reward task across the whole brain for our primary contrast of interest, win compared to loss trials. Whole brain analyses revealed greater bilateral activation during loss compared to win trials in the cuneus and anterior cingulate cortex (3dClustSim *p*_unc_ < .01, alpha < .05; S7 Table; S9 Fig).

#### Demographic characteristics

There were no significant clusters in the VS above *p*_unc_ < .01 associated with gender, age, and income-to-needs ratio. For pubertal development, right VS activation was greater during win compared to loss trials when controlling for gender (3dClustSim *p*_unc_ < .01, alpha < .05; Fig 7C).

## Discussion

The present study validated a child-friendly neuroimaging task in a large, community-based sample of youth with substantial enrichment for neighborhood poverty. We found that the task was engaging for youth of all ages, robustly activated the VS in response to reward, and VS activation did not differ according to reward magnitude. Advance knowledge of a prize based on task performance did not affect VS activation to the task, nor did pubertal development, gender, or income-to-needs ratio. There was a quadratic relationship between age and left VS activation during win compared to neutral trials in the MTwiNS sample. Importantly, we replicated main effects of the task in subsamples of genetically independent participants from MTwiNS and a younger sample of children from lower income families relative to the MTwiNS sample. Results from the present study demonstrate that this task can detect developmental differences in reward processing in cross-sectional samples of youth and is effective for studying reward processing in samples of youth from different socioeconomic backgrounds. Moreover, the short length of the task (approximately 10 minutes), engaging story, straightforward instructions, and inclusion of both win and loss trials make this task ideal for studying different aspects of reward processing in developmental samples of youth with a wide age range (e.g., 7–19 years).

### VS activation to reward

In two independent samples of youth, VS activation was greater during win compared to loss trials. Across the brain, the task activated key reward circuitry including the caudate, insula, nucleus accumbens, putamen, supplemental motor area, and thalamus. Differences in activation between win and loss trials could be attributable to variability in the neural response to winning, losing, or both. Thus, we also tested whether activation differed when comparing win and loss trials to neutral trials. In MTwiNS, there was greater VS activation to win compared to neutral trials. Across the whole brain, the task activated the insula, supplemental motor area, and thalamus during win and loss trials compared to neutral trials. These findings are consistent with recent meta-analyses [24, 25] and research on the neural response to reward in youth [41] and indicate that our task is an effective tool for studying reward circuitry in children and adolescents.

A growing concern in the field of developmental neuroscience is the reliability of task-evoked brain activation and its use as a tool to understand individual differences in behavior [42–44]. Notably, within-session reliability and longitudinal stability of brain activation to the MID task was poor in the ABCD Study, which challenges the notion that task-evoked brain activation is a stable and trait-like measure [44]. Without repeated measurements separated by days or weeks [42], we could not test the reliability of brain activation in response to our reward task over a short period of time. However, correlations in VS activation between complete twin pairs and trending associations for monozygotic twin pairs suggest there is at least some (small) reliable signal of VS activation in those data. Consistent with studies on short-term reliability of the neural response to reward in adults [45], the signal of VS activation seems to be more reliable within twin pairs during the total win compared to neutral contrast (i.e., when not combining the effects of both win and loss in a single contrast). Therefore, our study is contributing to the growing literature that suggests some contrasts, particularly those with a single active condition [42], may be more reliable than others. At the same time, the within family/twin pair correlation of this task was quite low in effect size. Thus, though there may be some, small, reliability in the signal, there is also substantial error/noise in these estimates. Future studies will need to be conducted to fully evaluate the reliability of our reward task and examine whether specific analytic approaches (e.g., multivariate analysis) may improve within twin pair correlation.

### Reward magnitude

Previous studies have demonstrated that youth are sensitive to reward magnitude, notably that the VS shows greater activation to larger monetary rewards during a combined anticipation and receipt phase [46]. However, we did not find a similar effect in our MTwiNS sample. Rather, the VS response to winning or losing large was the same as winning or losing small, respectively. Given we used a similar block design as Galván et al. [46], it may be that currency is a more salient reward than points for youth. Luking et al. [47] found that compared to adults, pre-pubertal aged children showed greater activation to loss in the insula. Thus, it may be that pre-pubertal aged children are particularly sensitive to gradations of loss and this sensitivity may reduce as children begin puberty. This explanation is consistent with our finding that VS activation was greater when losing large compared to small in the younger ABC Brains sample. Although reward magnitude was not associated with VS activation in the older MTwiNS sample, magnitude of reward was associated with other reward circuitry, notably the supplemental motor area and insula.

### Age, pubertal development, and gender associations with VS activation

The years spanning from early to late adolescence are key developmental periods for the VS, both in terms of structure and sensitivity to reward [28, 48]. Thus, we tested whether VS activation to reward was associated with age and pubertal development in two cross-sectional studies of youth ranging from 7 to 19 years old. Interestingly, only age was associated with VS activation to reward in the older MTwiNS sample. Pubertal development was not associated with VS activation to reward, nor was there an interaction between pubertal development and gender. The quadratic relationship between age and VS activation to reward followed a “U” shaped curve, with activation decreasing up until mid-adolescence, at which point it began to increase through late adolescence. These findings conflict with previous theory and research that suggest neural sensitivity to reward follows an inverted “U” shaped curve, with activation peaking in mid-adolescence before declining into adulthood [1, 4]. However, our age-related finding should be interpreted with caution as it did not hold up in the subsample of MTwiNS youth ages 10 to 18 years old. Instead, VS activation increased across adolescence in the age restricted MTwiNS subsample. Thus, there may be a shift in VS activation to reward from childhood to adolescence or alternatively, a small number of youth younger than 10 and older than 18 could be driving the “U” shaped effect, which could suggest that non-linear effects found across age may be due, in part, to children or young adults in the sample (whereas change during the teen years is linear).

Previous studies have also linked VS activation to reward to pubertal development and gonadal hormone levels in pubertal-aged youth [21–23]. In the younger ABC Brains sample, pubertal development was associated with right VS activation when comparing win and loss trials, though at a lower statistical threshold. Thus, more advanced pubertal development was associated with greater right VS activation. Conflicting results maybe be due to the smaller age range or younger age of children participating in ABC Brains (8–11 years, *M* = 10.23 years, *SD* = 0.91) compared to MTwiNS (7–19 years, *M* = 14.63 years, *SD* = 2.14). Moreover, the few studies that have examined maturational effects on reward processing were also cross-sectional and included small, homogenous samples. These relationships might not extend to larger and more socioeconomically diverse samples of youth [13]. Although the age effects in the MTwiNS sample suggest that this task is ideal for samples with a wide age range of youth, more research is needed with longitudinal studies.

Boys and girls did not differ in their VS response to reward. While research has shown that adult men and women recruit different neural networks in response to reward and differ in the strength of VS activation [49, 50], most studies of adolescents suggest no gender effect or use sample sizes too small to test for such an effect [49]. One recent study of 1,510 adolescents found gender differences in putamen activation during reward anticipation but not receipt [20]. A second study of 128 socioeconomically diverse adolescents found no gender differences in reward processing [51]. Thus, the lack of gender differences in VS activation to reward in two independent samples of youth, including one sample with a fairly large sample size, fits into the larger body of research on VS activation to reward during adolescence.

### Income-to-needs ratio and VS activation to reward

The vast majority of neuroimaging studies on reward processing are performed with convenience samples that generally represent well-educated, European-American youth [12–14]. It is not clear whether tasks that aim to activate reward circuitry will perform the same in samples with high rates of poverty and/or that include more substantial representation of ethnic and racial minority youth. Indeed, previous research has been conflicting, with adults from low socioeconomic status households showing decreased activation to reward [17], whereas children from low and middle socioeconomic status households did not differ in reward processing [52]. However, both studies included small samples. Thus, it was noteworthy that income-to-needs ratio was not associated with VS activation to reward in our relatively large sample of youth, even though our primary and replication samples had high rates of socioeconomic disadvantage and our primary sample had a large range of family incomes represented.

### Experimental prize condition

Interestingly, whether youth had knowledge of the prize before starting the task or after completing the task did not affect VS activation to reward. This outcome was surprising because most studies of reward processing use monetary incentives to encourage engagement with the task. Our finding suggests that task engagement was still high when only receiving points, even if youth did not expect to receive a physical prize.

### Limitations

The present study had many strengths, including a fairly large population-based sample of youth with substantial enrichment for neighborhood impoverishment, a younger replication sample with high rates of socioeconomic disadvantage, and an experimental prize condition. Furthermore, we are providing the task on our lab website for others to use. However, there were also limitations. First, because we do not have multiple measures of youths’ VS response to the reward task over short periods of time (i.e., days or weeks), we could not test whether the task is reliable. Positive correlations in VS activation to reward between all complete twin pairs and monozygotic twin pairs only suggest there may be some reliable signal of VS activation, particularly during the total win vs. neutral contrast. However, this within twin correlation was very small, and in many cases, not significant. Thus, there is likely substantial error/noise in these estimates as well. We are currently engaging the MTwiNS sample in a second wave of data collection 1–3 years later with a goal of reporting on the stability of activation to this task across longer periods during adolescence. Second, we used parental, rather than youth, report of youths’ pubertal development and included a wide age range of participants. Thus, it could be that we found no pubertal development effects in the VS in the MTwiNS sample due to our measurement of pubertal development and study design. However, previous studies have demonstrated that in the absence of ratings by a trained medical professional, parent ratings of youths’ pubertal development are acceptable, although may be more accurate for girls than boys [53, 54]. Finally, the high exclusion rate in our replication study resulted in a small sample size, which may lead to lower power and inconsistent estimates. However, the exclusion rate is comparable to other neuroimaging studies [55]. Even with a small sample, we were able to demonstrate that the task activated reward circuitry. Thus, not only is this task ideal for large-scale epidemiological studies, but also small-scale studies of youth.

### Conclusions

We found that a card-flipping reward task robustly activated the VS, a key region for reward processing, in two independent samples of youth ages 7 to 19. Youth in the two samples experienced high rates of socioeconomic disadvantage, and thus represent a population that is typically understudied in neuroimaging research. The task was able to detect age differences in VS activation, suggesting this task is suitable for large-scale, developmental studies of youth from all socioeconomic backgrounds. However, conflicting results for age and VS activation and lack of data on reliability indicates the need for further exploration. The short length (approximately 10 minutes) also makes this task ideal for studies wanting to investigate multiple neural circuits within the same study. We are making this task freely available on our lab website (https://sites.lsa.umich.edu/mindlab/research-projects/open-science/) so that future studies can continue to advance our knowledge on development of reward circuitry during childhood and adolescence.

## Supporting information

S1 Fig Age frequencies for youth participating in MTwiNS and the ABC Brains study. Age in years is reported for the MTwiNS (*n* = 464) and ABC Brains study (*n* = 27) youth included in fMRI analyses.
(TIFF)

S2 Fig Whole brain activation that overlaps with the ventral striatum mask during a child-friendly reward task in the MTwiNS sample (*n* = 446). k = number of voxels within the cluster that overlap with the ventral striatum mask. **(a) Total win > total loss trials.** Left: k = 100, Right: k = 76. **(b) Total win > neutral trials.** Left: k = 4.
(TIF)

S3 Fig Whole brain activation during (a) total win > neutral trials and (b) neutral > total win trials during a child-friendly reward task in the MTwiNS sample (*n* = 446). k = number of voxels within the cluster. False positive rate is controlled using 3dClustSim for cluster-level correction (punc < .001, alpha < .05, k > 57).
(TIF)

S4 Fig Whole brain activation during total loss > neutral trials during a child-friendly reward task in the MTwiNS sample (*n* = 446). k = number of voxels within the cluster. False positive rate is controlled using 3dClustSim for cluster-level correction (p_unc_ < .001, alpha < .05, k > 57).
(TIF)

S5 Fig Whole brain activation during neutral > total loss trials during a child-friendly reward task in the MTwiNS sample (*n* = 446). k = number of voxels within the cluster. False positive rate is controlled using 3dClustSim for cluster-level correction (p_unc_ < .001, alpha < .05, k > 57).
(TIF)

S6 Fig Whole brain activation during (a) large win > small win trials and (b) small win > large win trials in the MTwiNS sample (*n* = 446). k = number of voxels within the cluster. False positive rate is controlled using 3dClustSim for cluster-level correction (p_unc_ < .001, alpha < .05, k > 57).
(TIF)

S7 Fig Whole brain activation during (a) large loss > small loss trials and (b) small loss > large loss trials in the MTwiNS sample (*n* = 446). k = number of voxels within the cluster. False positive rate is controlled using 3dClustSim for cluster-level correction (p_unc_ < .001, alpha < .05, k > 57).
(TIF)

S8 Fig Activation is greater for youth in the before prize condition (*n* = 226) vs. after prize condition (*n* = 220) during large win > small win trials in the MTwiNS sample (*n* = 446). k = number of voxels within the cluster. Left: k = 72, T = 4.25, MNI -24, -96, 2. False positive rate is controlled using 3dClustSim for cluster-level correction (p_unc_ < .001, alpha < .05, k > 57).
(TIF)

S9 Fig Whole brain activation during total loss > total win trials during a child-friendly reward task in the ABC Brains sample (*n* = 21). False positive rate is controlled using 3dClustSim for cluster-level correction (*p*_unc_ < .01, alpha < .05, k > 348).
(TIF)

S1 Table Summary of exclusion criteria. ^a^For MTwiNS, participants were excluded if their response rate to the task was less than 80%. For ABC Brains, the response rate threshold was increased to 90% because we were unable to monitor participants’ task engagement with an eye tracker. ^b^Participants were excluded if ventral striatum coverage was less than 70% for MTwiNS and less than 90% for ABC Brains. The MTwiNS threshold was lower due to increased susceptibility to artifacts associated with multiband echo-planar imaging acquisition.
(DOCX)

S2 Table Main effects of task and associations with age in the ventral striatum in MTwiNS youth between the ages of 10 and 18 years old. *n* = 454. k = number of voxels within the cluster. MTwiNS youth were excluded from analyses if they were younger than 10 (*n* = 9) or older than 18 years old (*n* = 1) to confirm that our results were not due to ages at the extreme ends of the distribution. Significant clusters were identified in SPM12 using a mask of the ventral striatum [9], grey matter segmented using AFNI [36]. False positive rate is controlled across the ventral striatum using 3dClustSim for cluster-level correction (*p*_unc_ < .001, alpha < .05, k > 3).
(DOCX)

S3 Table Correlations of extracted ventral striatum activation between complete twin pairs from the MTwiNS sample. VS = ventral striatum. k = number of voxels within the cluster. VS activation during total win > total loss trials and total win > neutral trials was extracted and correlated between all complete twin pairs, monozygotic twin pairs only, and dizygotic twin pairs only.
(DOCX)

S4 Table MTwiNS whole brain main effects of task for total win and total loss compared to neutral. *n* = 446. k = number of voxels within the cluster. False positive rate is controlled for using 3dClustSim for cluster-level correction (*p*_unc_ < .001, alpha < .05, k > 57). Anatomical region labels were retrieved from the AAL3 atlas [40]. The anatomical regions listed are not exhaustive, but full activation can be seen in S3–S5 Figs for full slices.
(DOCX)

S5 Table MTwiNS whole brain results for magnitude of reward. *n* = 446. k = number of voxels within the cluster. False positive rate is controlled for using 3dClustSim for cluster-level correction (*p*_unc_ < .001, alpha < .05, k > 57). Anatomical region labels were retrieved from the AAL3 atlas [40]. The anatomical regions listed are not exhaustive, but full activation can be seen in S6 and S7 Figs for full slices.
(DOCX)

S6 Table Main effects of task in the ventral striatum in 10 genetically independent subsamples of MTwiNS youth. *n* = 277. k = number of voxels within the cluster. To confirm results were not impacted by the nesting of twins within families, we tested for main effects of task in the ventral striatum in 10 genetically independent subsamples of MTwiNS youth. The genetically independent subsamples included all twins who did not have a co-twin scanned (*n* = 90) and a randomly selected twin from all complete twin pairs (*n* = 187). Significant clusters were identified in SPM12 using a mask of the ventral striatum [9], grey matter segmented using AFNI [36]. False positive rate is controlled across the ventral striatum using 3dClustSim for cluster-level correction (*p*_unc_ < .001, alpha < .05, k > 3). ^a^One cluster was trending at a lower statistical threshold (*p*_unc_ < .001, alpha < .10, k > 2).
(DOCX)

S7 Table Brain regions showing greater activation to total win vs. total loss in the ABC Brains sample. *n* = 21. k = number of voxels within the cluster. False positive rate is controlled across the whole brain using 3dClustSim for cluster-level correction (p_unc_ < .01, alpha < .05, k > 348). Anatomical region labels were retrieved from the AAL3 atlas [40]. The anatomical regions listed are not exhaustive, but full activation can be seen in S9 Fig for full slices.
(DOCX)
