# Supplementary figures and images for: A brief child-friendly reward task reliably activates the ventral striatum in two samples of socioeconomically diverse youth
Source: PLoS One. 2022 Feb 3;17(2):e0263368. doi: 10.1371/journal.pone.0263368 (PMC8812963; doi:10.1371/journal.pone.0263368)

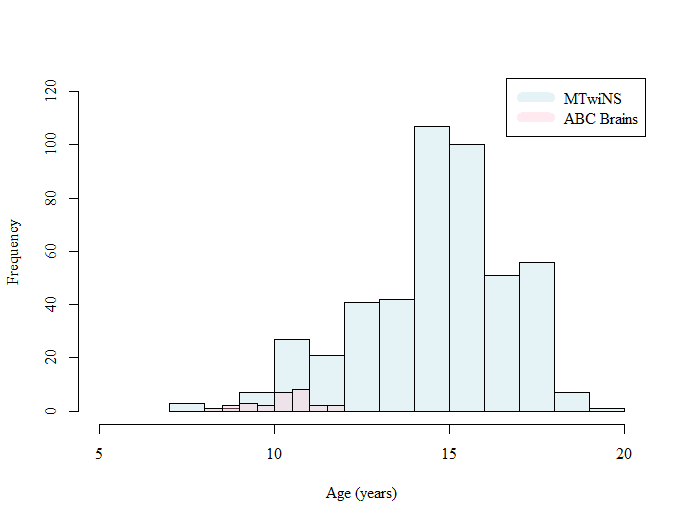

Supplement: S1 Fig — Age in years is reported for the MTwiNS (n = 464) and ABC Brains study (n = 27) youth included in fMRI analyses. (TIFF) [file pone.0263368.s001.tiff]

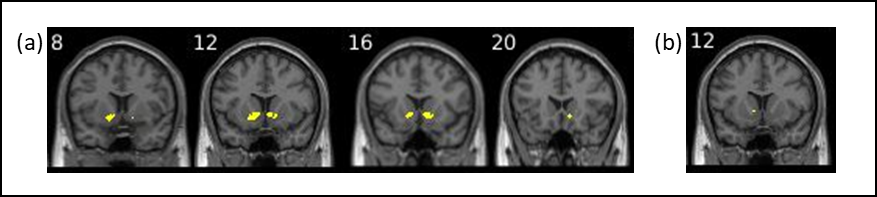

Supplement: S2 Fig — k = number of voxels within the cluster that overlap with the ventral striatum mask. (a) Total win > total loss trials. Left: k = 100, Right: k = 76. (b) Total win > neutral trials. Left: k = 4. (TIF) [file pone.0263368.s002.tif]

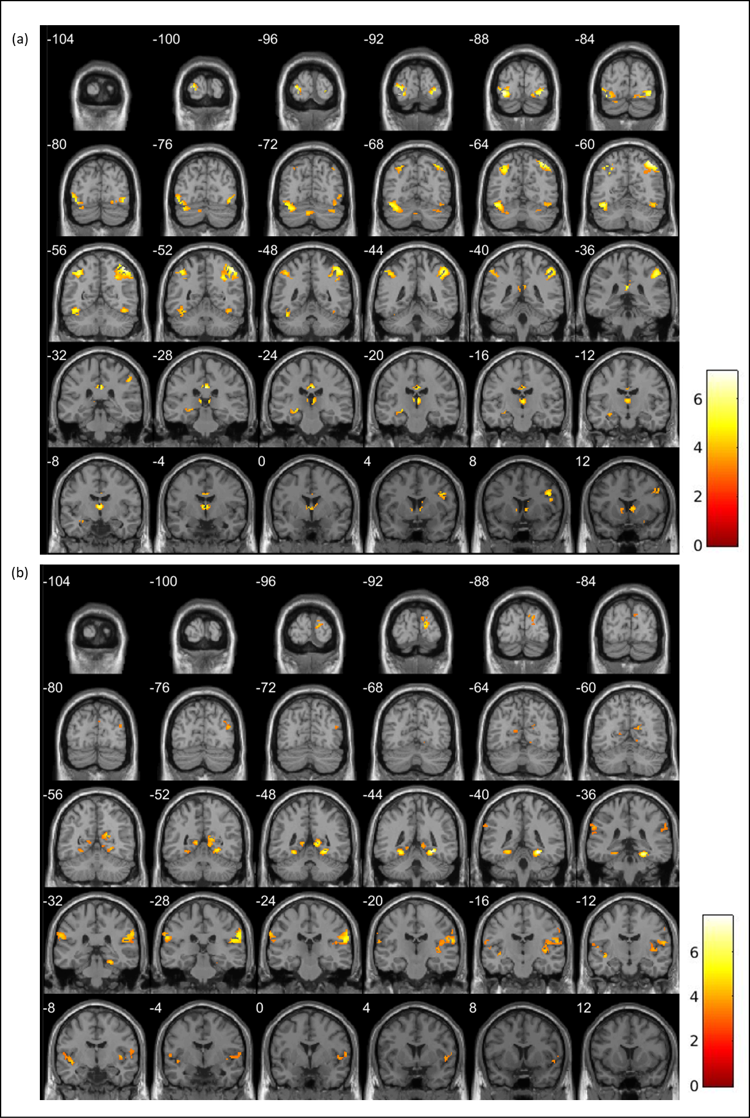

Supplement: S3 Fig — Whole brain activation during (a) total win > neutral trials and (b) neutral > total win trials during a child-friendly reward task in the MTwiNS sample (n = 446). k = number of voxels within the cluster. False positive rate is controlled using 3dClustSim for cluster-level correction (punc < .001, alpha < .05, k > 57). (TIF) [file pone.0263368.s003.tif]

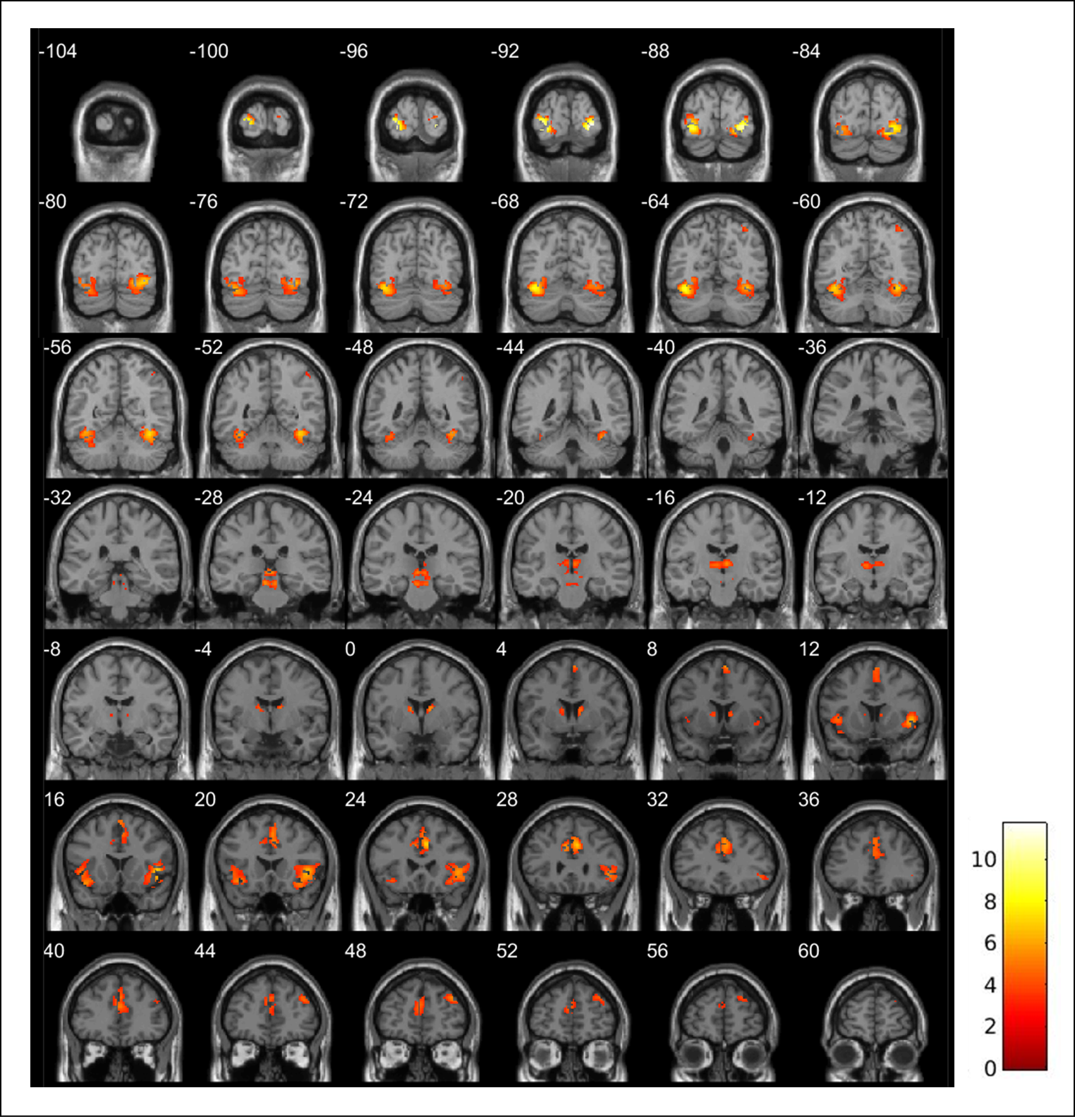

Supplement: S4 Fig — k = number of voxels within the cluster. False positive rate is controlled using 3dClustSim for cluster-level correction (punc < .001, alpha < .05, k > 57). (TIF) [file pone.0263368.s004.tif]

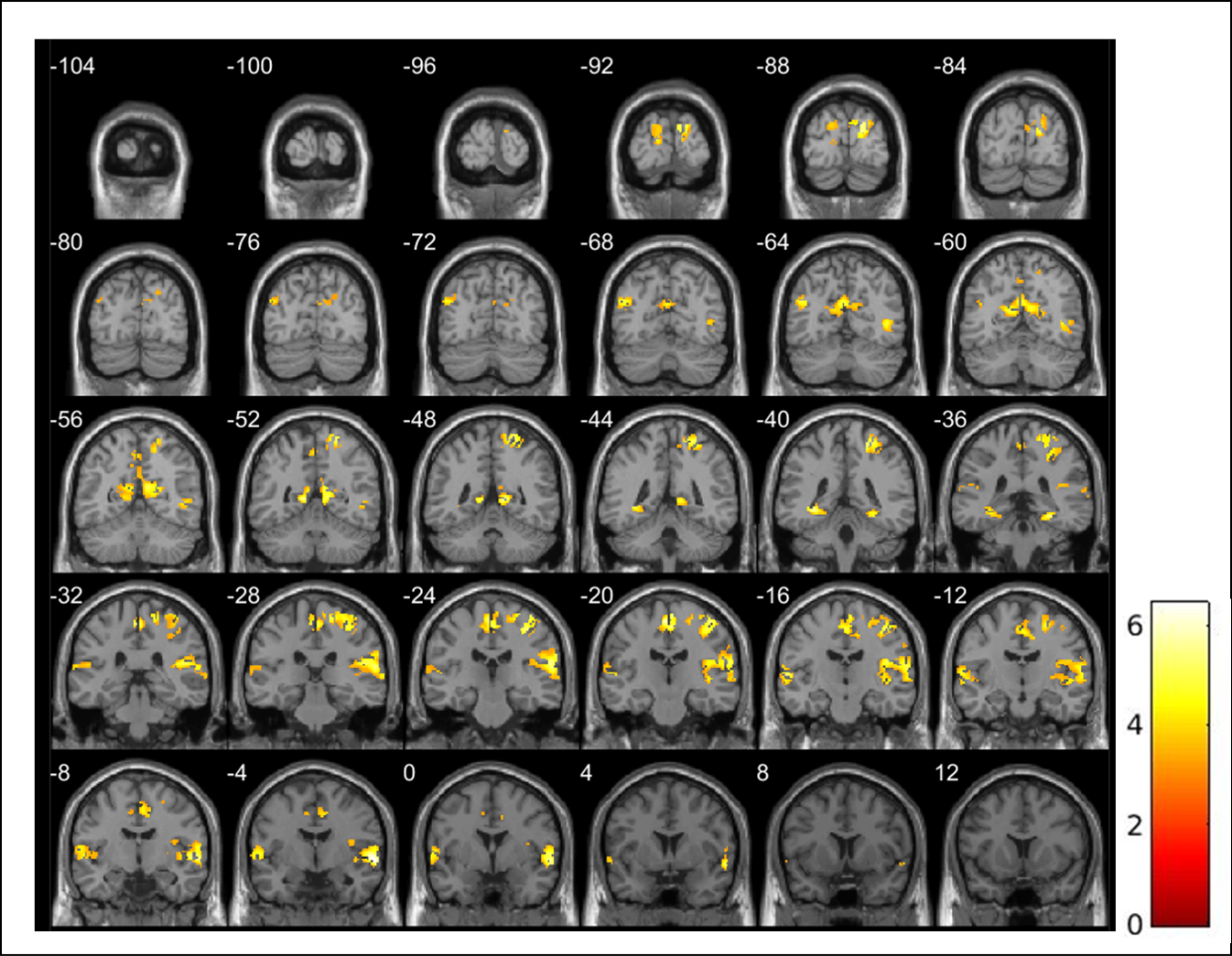

Supplement: S5 Fig — k = number of voxels within the cluster. False positive rate is controlled using 3dClustSim for cluster-level correction (punc < .001, alpha < .05, k > 57). (TIF) [file pone.0263368.s005.tif]

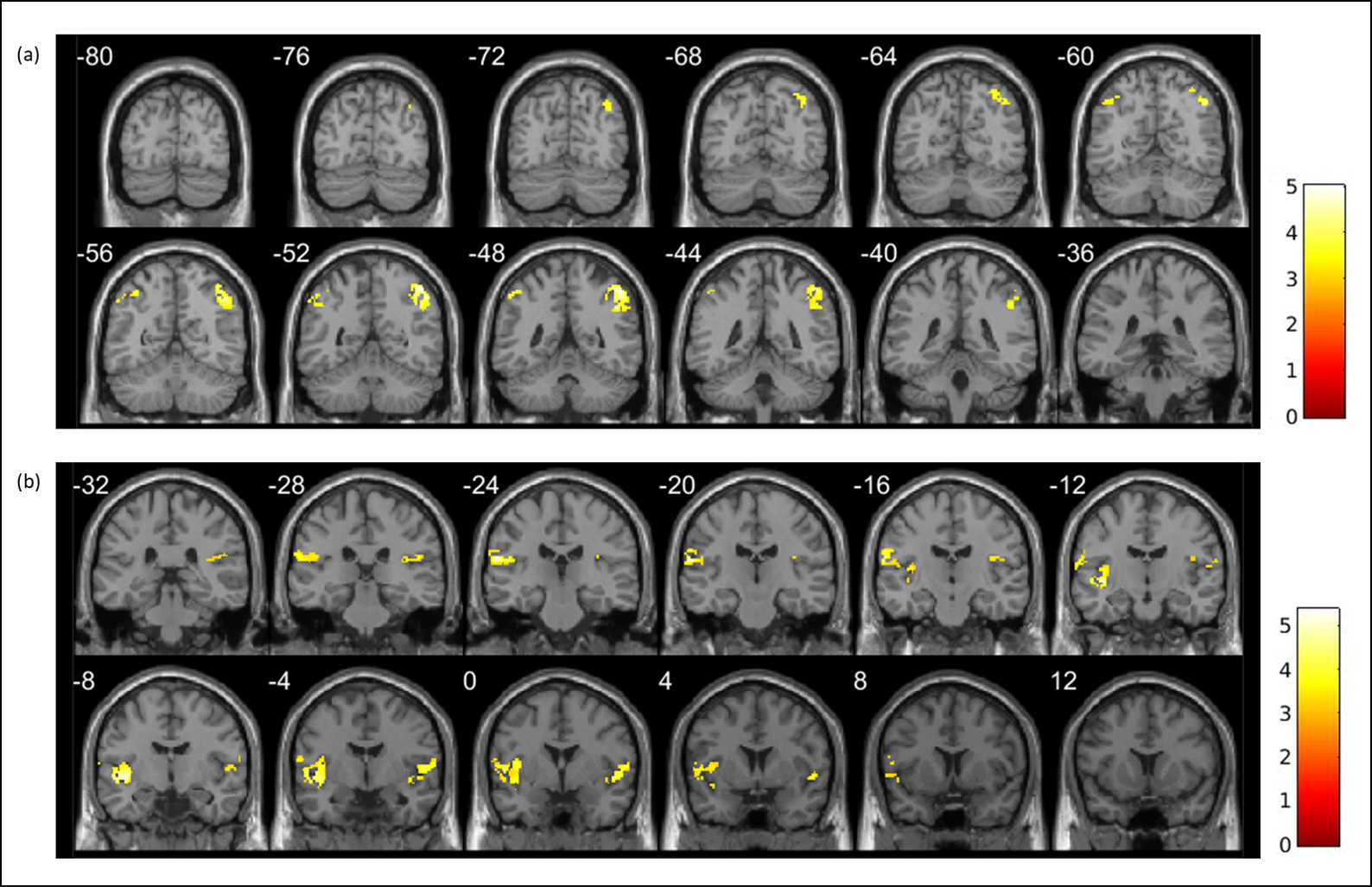

Supplement: S6 Fig — Whole brain activation during (a) large win > small win trials and (b) small win > large win trials in the MTwiNS sample (n = 446). k = number of voxels within the cluster. False positive rate is controlled using 3dClustSim for cluster-level correction (punc < .001, alpha < .05, k > 57). (TIF) [file pone.0263368.s006.tif]

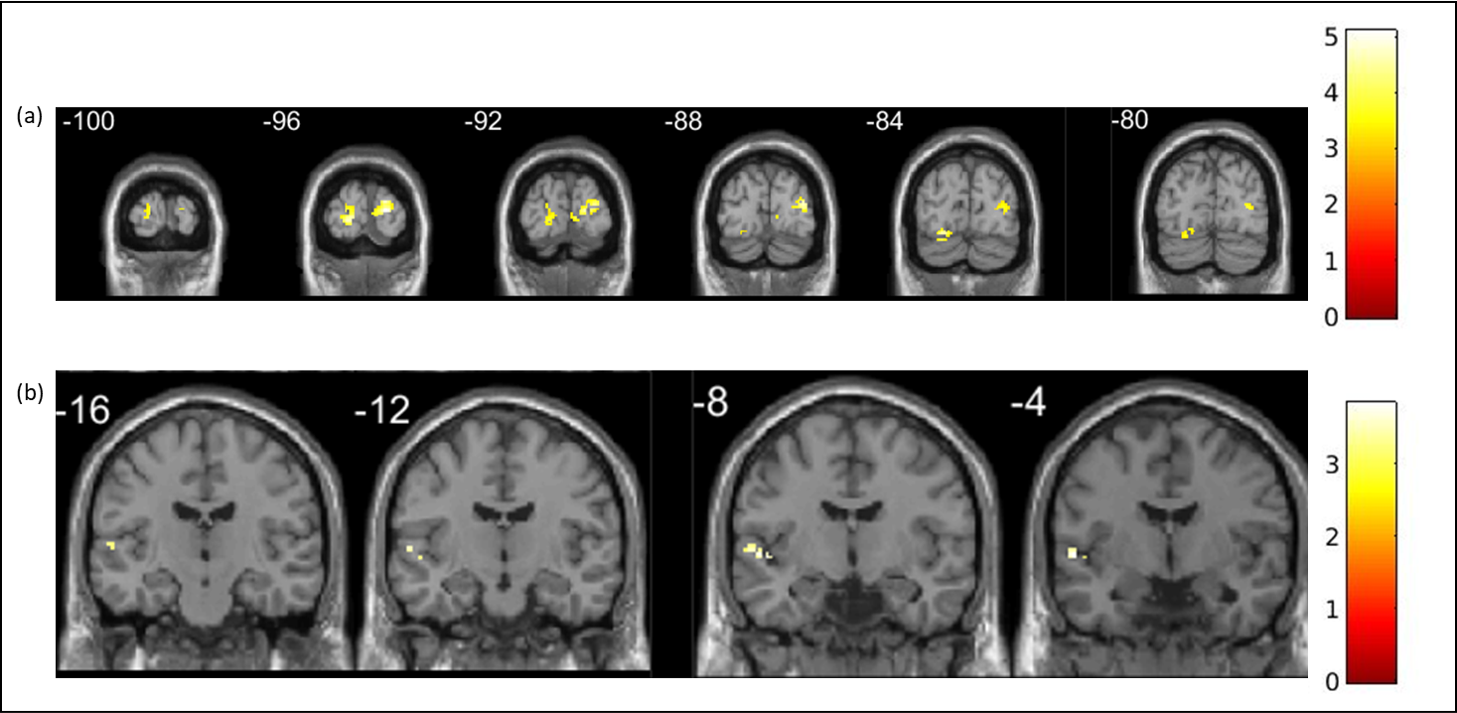

Supplement: S7 Fig — Whole brain activation during (a) large loss > small loss trials and (b) small loss > large loss trials in the MTwiNS sample (n = 446). k = number of voxels within the cluster. False positive rate is controlled using 3dClustSim for cluster-level correction (punc < .001, alpha < .05, k > 57). (TIF) [file pone.0263368.s007.tif]

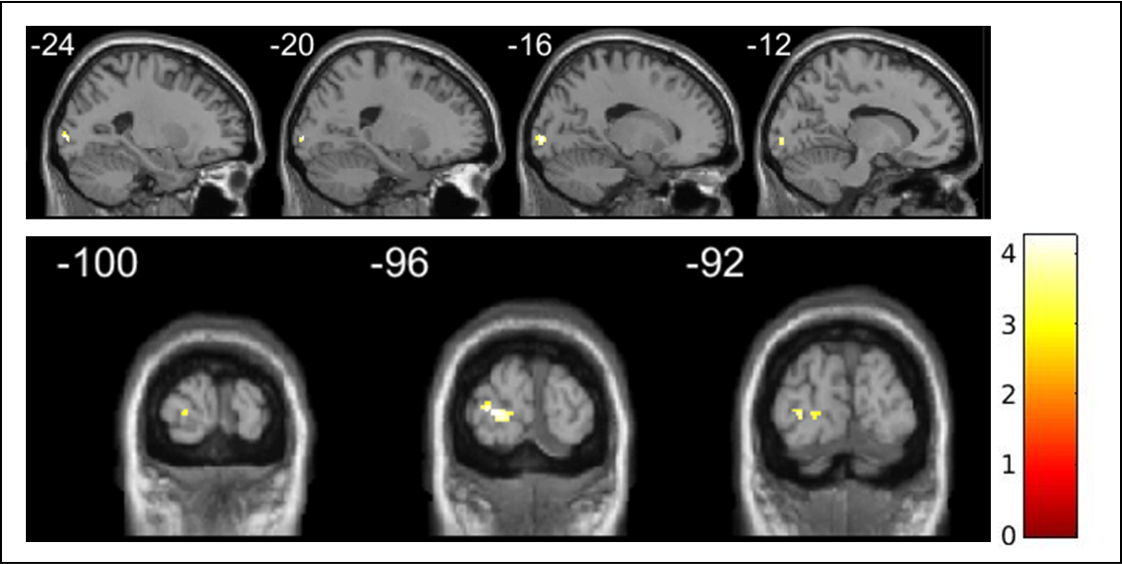

Supplement: S8 Fig — k = number of voxels within the cluster. Left: k = 72, T = 4.25, MNI -24, -96, 2. False positive rate is controlled using 3dClustSim for cluster-level correction (punc < .001, alpha < .05, k > 57). (TIF) [file pone.0263368.s008.tif]

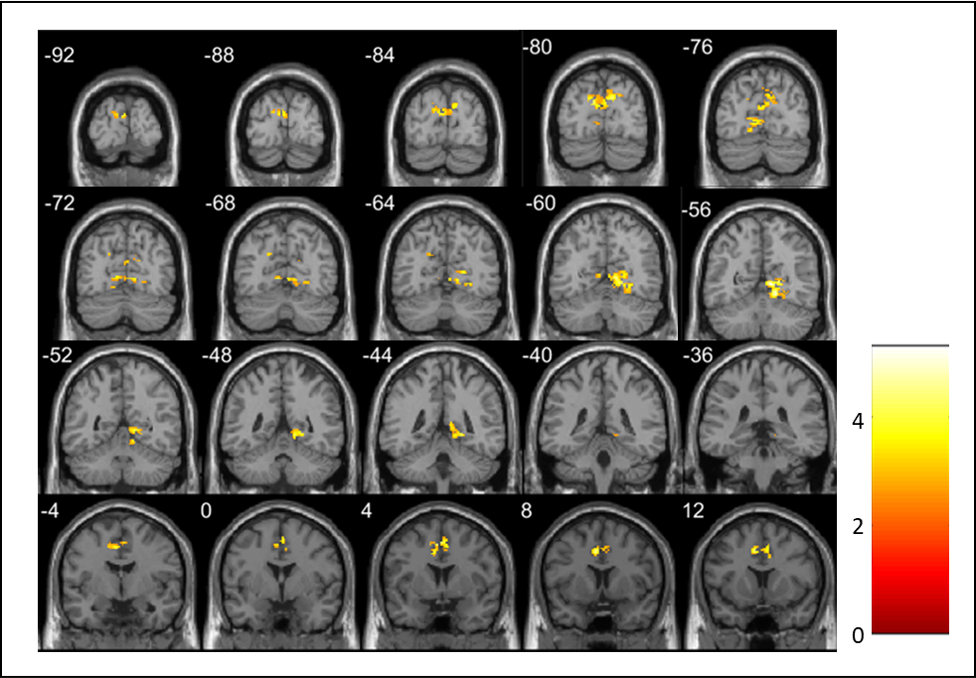

Supplement: S9 Fig — False positive rate is controlled using 3dClustSim for cluster-level correction (punc < .01, alpha < .05, k > 348). (TIF) [file pone.0263368.s009.tif]
