# Supplementary material for: A brief child-friendly reward task reliably activates the ventral striatum in two samples of socioeconomically diverse youth
Source: PLoS One. 2022 Feb 3;17(2):e0263368. doi: 10.1371/journal.pone.0263368 (PMC8812963; doi:10.1371/journal.pone.0263368)
Supplement: S1 Table — aFor MTwiNS, participants were excluded if their response rate to the task was less than 80%. For ABC Brains, the response rate threshold was increased to 90% because we were unable to monitor participants’ task engagement with an eye tracker. bParticipants were excluded if ventral striatum coverage was less than 70% for MTwiNS and less than 90% for ABC Brains. The MTwiNS threshold was lower due to increased susceptibility to artifacts associated with multiband echo-planar imaging acquisition. (DOCX) [file pone.0263368.s010.docx]

S1 Table. Summary of exclusion criteria

|  | MTwiNS | | ABC Brains | |
| --- | --- | --- | --- | --- |
| Exclusion Criteria | Number  Excluded | Sample  Size | Number  Excluded | Sample  Size |
| Total number of participants |  | 708 |  | 56 |
| Participant discontinued study before completing reward task | 112 |  | 13 |  |
| Missing behavioral data | 6 |  | 2 |  |
| Major medical or neurological disorder | 18 |  | 0 |  |
| Excessive movement | 1 |  | 2 |  |
| Low response rate during task^a^ | 54 |  | 10 |  |
| Low coverage of ventral striatum^b^ | 53 |  | 2 |  |
| Final Sample | 244 | 464 | 29 | 27 |
