# Supplementary material for: A brief child-friendly reward task reliably activates the ventral striatum in two samples of socioeconomically diverse youth
Source: PLoS One. 2022 Feb 3;17(2):e0263368. doi: 10.1371/journal.pone.0263368 (PMC8812963; doi:10.1371/journal.pone.0263368)
Supplement: S2 Table — n = 454. k = number of voxels within the cluster. MTwiNS youth were excluded from analyses if they were younger than 10 (n = 9) or older than 18 years old (n = 1) to confirm that our results were not due to ages at the extreme ends of the distribution. Significant clusters were identified in SPM12 using a mask of the ventral striatum [9], grey matter segmented using AFNI [36]. False positive rate is controlled across the ventral striatum using 3dClustSim for cluster-level correction (punc < .001, alpha < .05, k > 3). (DOCX) [file pone.0263368.s011.docx]

S2 Table. Main effects of task and associations with age in the ventral striatum in MTwiNS youth between the ages of 10 and 18 years old

| Analysis | Contrast | Side | Peak (x, y, z) | T | k |
| --- | --- | --- | --- | --- | --- |
| Main Effect of Task | Total Win > Total Loss | Left | -12, 10, -6 | 4.10 | 83 |
|  |  | Right | 12, 14, -4 | 4.03 | 86 |
|  | Total Win > Neutral | Left | -8, 12, -2 | 3.18 | 4 |
| Age | Total Win > Neutral | Left | -22, 12, -10 | 3.70 | 6 |
