# Supplementary material for: A brief child-friendly reward task reliably activates the ventral striatum in two samples of socioeconomically diverse youth
Source: PLoS One. 2022 Feb 3;17(2):e0263368. doi: 10.1371/journal.pone.0263368 (PMC8812963; doi:10.1371/journal.pone.0263368)
Supplement: S3 Table — VS = ventral striatum. k = number of voxels within the cluster. VS activation during total win > total loss trials and total win > neutral trials was extracted and correlated between all complete twin pairs, monozygotic twin pairs only, and dizygotic twin pairs only. (DOCX) [file pone.0263368.s012.docx]

S3 Table. Correlations of extracted ventral striatum activation between complete twin pairs from the MTwiNS sample

|  |  | Total Win > Total Loss | | | | Total Win > Neutral | | | | | |
| --- | --- | --- | --- | --- | --- | --- | --- | --- | --- | --- | --- |
|  |  | Left VS  (k = 77) | | Right VS  (k = 72) | | Left VS  (k = 12) | | Right VS Cluster 1  (k = 6) | | Right VS Cluster 2  (k = 3) | |
| Complete Twin Pairs | *n* (pairs) | *r* | *p* | *r* | *p* | *r* | *p* | *r* | *p* | *r* | *p* |
| All complete twin pairs | 187 | .036 | .480 | .087 | .090 | .010 | .840 | .110 | .040 | .020 | .746 |
| Monozygotic twin pairs only | 77 | .033 | .690 | .075 | .350 | .160 | .051 | .144 | .075 | .050 | .544 |
| Dizygotic twin pairs only | 110 | .038 | .570 | .099 | .140 | -.064 | .348 | .078 | .251 | -.007 | .921 |
