# Supplementary material for: A brief child-friendly reward task reliably activates the ventral striatum in two samples of socioeconomically diverse youth
Source: PLoS One. 2022 Feb 3;17(2):e0263368. doi: 10.1371/journal.pone.0263368 (PMC8812963; doi:10.1371/journal.pone.0263368)
Supplement: S4 Table — n = 446. k = number of voxels within the cluster. False positive rate is controlled for using 3dClustSim for cluster-level correction (punc < .001, alpha < .05, k > 57). Anatomical region labels were retrieved from the AAL3 atlas [40]. The anatomical regions listed are not exhaustive, but full activation can be seen in S3–S5 Figs for full slices. (DOCX) [file pone.0263368.s013.docx]

S4 Table. MTwiNS whole brain main effects of task for total win and total loss compared to neutral

| Contrast | Side | Region Labels | Peak (x,y,z) | T | k |
| --- | --- | --- | --- | --- | --- |
| Total Win > Neutral | Right | Superior parietal gyrus, Inferior parietal gyrus, Angular gyrus | 42, -58, 54 | 7.14 | 1238 |
|  | Left | Inferior occipital gyrus, Superior occipital gyrus, Lingual gyrus, Fusiform gyrus | -30, -90, -8 | 7.00 | 1143 |
|  | Right | Middle cingulate &  paracingulate gyri, Posterior cingulate gyrus | 2, -26, 32 | 6.39 | 235 |
|  | Left | Middle cingulate &  paracingulate gyri, Posterior cingulate gyrus |  |  |  |
|  | Left | Inferior parietal gyrus | -36, -56, 52 | 6.19 | 508 |
|  | Right | Thalamus | 2, -20, 8 | 5.98 | 456 |
|  | Left | Thalamus |  |  |  |
|  | Right | Middle occipital gyrus, Superior occipital gyrus, Fusiform gyrus, Lingual gyrus | 36, -86, -6 | 5.98 | 550 |
|  | Right | Middle frontal gyrus, Inferior frontal gyrus (triangular part) | 48, 42, 20 | 5.41 | 121 |
|  | Right | Precentral gyrus, Frontal inferior operculum | 46, 6, 32 | 5.09 | 213 |
|  | Left | Hippocampus, Thalamus, Putamen | -26, -22, -8 | 4.92 | 118 |
|  | Left | Vermis, Cerebellum | -4, -72, -30 | 4.25 | 73 |
|  | Right | Insula, Putamen | 36, 18, -4 | 4.21 | 128 |
|  | Left | Supplementary motor area, Superior frontal gyrus (medial), Cingulate | 4, 20, 46 | 4.15 | 107 |
|  | Right | Supplementary motor area, Superior frontal gyrus (medial), |  |  |  |
|  | Right | Cerebellum, Fusiform gyrus | 34, -72, -26 | 3.84 | 58 |
|  | Left | Anterior cingulate cortex | 4, 42, 6 | 3.71 | 62 |
|  | Right | Anterior cingulate cortex |  |  |  |
| Neutral > Total Win | Right | Fusiform gyrus, Parahippocampal gyrus | 28, -42, -10 | 7.62 | 366 |
|  | Left | Fusiform gyrus, Parahippocampal gyrus | -26, -44, -12 | 5.77 | 308 |
|  | Right | Superior temporal gyrus, Rolandic operculum, Insula | 60, -26, 24 | 5.69 | 1108 |
|  | Right | Lingual gyrus, Precuneus, Calcarine fissure and surrounding cortex | 12, -48, 4 | 5.55 | 262 |
|  | Right | Calcarine fissure and surrounding cortex, Cuneus | 14, -94, 10 | 5.02 | 167 |
|  | Left | SupraMarginal gyrus, Insula, Superior temporal gyrus | -60, -28, 26 | 4.89 | 466 |
|  | Right | Middle occipital gyrus | 42, -78, 22 | 4.28 | 68 |
| Total Loss > Neutral | Right | Inferior occipital gyrus, Middle occipital gyrus, Lingual gyrus, Fusiform gyrus | 32, -92, -2 | 11.70 | 1596 |
|  | Left | Inferior occipital gyrus, Middle occipital gyrus, Lingual gyrus, Fusiform gyrus | -22, -96, -4 | 10.35 | 1651 |
|  | Right | Middle cingulate and paracingulate gyri, Anterior cingulate cortex, Supplementary motor area | 8, 24, 34 | 7.02 | 1234 |
|  | Left | Middle cingulate and paracingulate gyri, Supplementary motor area, Anterior cingulate cortex |  |  |  |
|  | Right | Insula, Inferior frontal gyrus (triangular part), Inferior frontal gyrus pars orbitalis | 46, 16, 2 | 6.94 | 908 |
|  | Right | Caudate | 12, 0, 16 | 5.49 | 99 |
|  | Right | Thalamus, Raphe, Vermis | 2, -28, -16 | 5.48 | 334 |
|  | Left | Thalamus |  |  |  |
|  | Right | Thalamus | 6, -16, 8 | 5.47 | 308 |
|  | Left | Thalamus |  |  |  |
|  | Left | Insula, Inferior frontal gyrus pars orbitalis | -40, 16, -12 | 5.35 | 437 |
|  | Right | Middle frontal gyrus, Superior frontal gyrus (dorsolateral) | 38, 48, 30 | 5.22 | 189 |
|  | Left | Caudate | -12, 0, 14 | 4.73 | 85 |
|  | Right | Inferior partietal gyrus, Superior parietal gyrus | 36, -62, 54 | 4.45 | 62 |
| Neutral > Total Loss | Right | Superior temporal gyrus | 60, -4, -2 | 6.46 | 1870 |
|  | Right | Superior occipital gyrus, Cuneus, Lingual gyrus, Precuneus | 16, -90 28 | 5.98 | 1327 |
|  | Left | Lingual Gyrus, Calcarine fissure and  surrounding cortex |  |  |  |
|  | Left | Paracentral lobule, Supplementary motor area | -4, -22, 62 | 5.95 | 683 |
|  | Right | Supplementary motor area, Paracentral lobule |  |  |  |
|  | Left | Hippocampus, Parahippocampal gyrus | -32, -40, -6 | 5.59 | 141 |
|  | Right | Superior parietal gyrus, Postcentral gyrus, Precentral gyrus | 22, -50, 66 | 5.43 | 1254 |
|  | Right | Fusiform gyrus, Parahippocampal gyrus | 28, -38, -12 | 5.27 | 84 |
|  | Left | Superior temporal gyrus, Middle temporal gyrus, Rolandic operculum | -58, -16, 6 | 5.17 | 603 |
|  | Left | Angular gyrus | -46, -66, 26 | 4.92 | 228 |
|  | Right | Middle temporal gyrus | 42, -64, 4 | 4.79 | 206 |
|  | Left | Superior occipital gyrus, Calcarine fissure and surrounding cortex, Cuneus | -12, -92, 10 | 4.29 | 127 |
|  | Left | Precuneus | -2, -54, 52 | 4.17 | 65 |
|  | Right | Precuneus |  |  |  |
|  | Left | Superior frontal gyrus (dorsolateral),  Middle frontal gyrus | -20, 24, 42 | 3.73 | 72 |
