# Supplementary material for: A brief child-friendly reward task reliably activates the ventral striatum in two samples of socioeconomically diverse youth
Source: PLoS One. 2022 Feb 3;17(2):e0263368. doi: 10.1371/journal.pone.0263368 (PMC8812963; doi:10.1371/journal.pone.0263368)
Supplement: S5 Table — n = 446. k = number of voxels within the cluster. False positive rate is controlled for using 3dClustSim for cluster-level correction (punc < .001, alpha < .05, k > 57). Anatomical region labels were retrieved from the AAL3 atlas [40]. The anatomical regions listed are not exhaustive, but full activation can be seen in S6 and S7 Figs for full slices. (DOCX) [file pone.0263368.s014.docx]

S5 Table. MTwiNS whole brain results for magnitude of reward

| Contrast | Side | Region Labels | Peak (x,y,z) | T | k |
| --- | --- | --- | --- | --- | --- |
| Large Win > Small Win | Right | Inferior parietal gyrus, Superior parietal gyrus | 46, -52, 54 | 5.02 | 703 |
|  | Right | Superior frontal gyrus (medial), Middle cingulate & paracingulate gyri | 2, 36, 40 | 4.37 | 121 |
|  | Left | Superior frontal gyrus (medial), Middle cingulate & paracingulate gyri |  |  |  |
|  | Right | Middle frontal gyrus | 44, 24, 40 | 4.33 | 137 |
|  | Left | Inferior parietal gyrus | -46, -48, 52 | 4.14 | 117 |
|  | Right | Superior frontal gyrus (medial), Supplementary motor area | 4, 26, 48 | 4.12 | 72 |
|  | Left | Superior frontal gyrus (medial), Supplementary motor area |  |  |  |
| Small Win > Large Win | Left | Insula, Superior temporal gyrus | -42, -10, -2 | 5.38 | 688 |
|  | Left | Postcentral gyrus, SupraMarginal gyrus | -60, -22, 20 | 4.98 | 359 |
|  | Right | Rolandic operculum, Superior temporal gyrus, Insula | 54, -4, 6 | 4.21 | 226 |
|  | Right | Superior temporal gyrus, Rolandic operculum, SupraMarginal gyrus | 48, -30, 20 | 3.63 | 100 |
| Large Loss > Small Loss | Right | Calcarine fissure and  surrounding cortex, Cuneus | 20, -96, 6 | 5.11 | 265 |
|  | Left | Calcarine fissure and  surrounding cortex, Middle occipital gyrus, Superior Occipital gyrus | -12, -98, 0 | 4.70 | 141 |
|  | Left | Lingual gyrus, Fusiform gyrus | -20, -84, -14 | 4.50 | 77 |
| Small Loss > Large Loss | Left | Superior temporal gyrus, Rolandic operculum | -54, -4, 0 | 3.84 | 61 |
