# Supplementary material for: A brief child-friendly reward task reliably activates the ventral striatum in two samples of socioeconomically diverse youth
Source: PLoS One. 2022 Feb 3;17(2):e0263368. doi: 10.1371/journal.pone.0263368 (PMC8812963; doi:10.1371/journal.pone.0263368)
Supplement: S6 Table — n = 277. k = number of voxels within the cluster. To confirm results were not impacted by the nesting of twins within families, we tested for main effects of task in the ventral striatum in 10 genetically independent subsamples of MTwiNS youth. The genetically independent subsamples included all twins who did not have a co-twin scanned (n = 90) and a randomly selected twin from all complete twin pairs (n = 187). Significant clusters were identified in SPM12 using a mask of the ventral striatum [9], grey matter segmented using AFNI [36]. False positive rate is controlled across the ventral striatum using 3dClustSim for cluster-level correction (punc < .001, alpha < .05, k > 3). aOne cluster was trending at a lower statistical threshold (punc < .001, alpha < .10, k > 2). (DOCX) [file pone.0263368.s015.docx]

S6 Table. Main effects of task in the ventral striatum in 10 genetically independent subsamples of MTwiNS youth

| Contrast | Subsample Number | Side | Peak (x, y, z) | T | k |
| --- | --- | --- | --- | --- | --- |
| Total Win > Total Loss | 2 | Right | 10, 16, -4 | 3.59 | 28 |
|  | 4 | Left | -12, 12, -6 | 3.58 | 32 |
|  |  | Right | 10, 16, -4 | 4.20 | 45 |
|  | 6 | Left | -6, 12, -2 | 3.31 | 13 |
|  |  | Right | 12, 14, -4 | 3.95 | 67 |
|  | 7 | Left | -12, 12, -6 | 3.34 | 9 |
|  | 8 | Right | 6, 12, -2 | 3.60 | 17 |
|  | 10 | Left | -12, 10, -6 | 3.67 | 25 |
|  |  | Right | 6, 12, -2 | 4.03 | 44 |
| Total Win > Neutral | 1 | Right | 14, 12, -4 | 3.46 | 10 |
|  | 4 | Left | -8, 12, -4 | 3.69 | 42 |
|  |  | Right | 10, 12, -2 | 3.25 | 4 |
|  | 6 | Right | 14, 12, -4 | 3.62 | 19 |
|  | 7 | Left | -8, 10, -2 | 3.13 | 2^a^ |
|  | 10 | Right | 8, 10, -2 | 3.49 | 3 |
|  |  |  | 14, 10, -2 | 3.37 | 3 |
