# Supplementary material for: A brief child-friendly reward task reliably activates the ventral striatum in two samples of socioeconomically diverse youth
Source: PLoS One. 2022 Feb 3;17(2):e0263368. doi: 10.1371/journal.pone.0263368 (PMC8812963; doi:10.1371/journal.pone.0263368)
Supplement: S7 Table — n = 21. k = number of voxels within the cluster. False positive rate is controlled across the whole brain using 3dClustSim for cluster-level correction (punc < .01, alpha < .05, k > 348). Anatomical region labels were retrieved from the AAL3 atlas [40]. The anatomical regions listed are not exhaustive, but full activation can be seen in S9 Fig for full slices. (DOCX) [file pone.0263368.s016.docx]

S7 Table. Brain regions showing greater activation to total win vs. total loss in the ABC Brains sample

| Contrast | Side | Region Labels | Peak (x,y,z) | T | k |
| --- | --- | --- | --- | --- | --- |
| Total Loss > Total Win | Right | Lingual gyrus, Vermis | 10, -58, 2 | 5.31 | 786 |
|  | Left | Anterior cingulate cortex | 0, 26, 18 | 4.76 | 415 |
|  | Right | Anterior cingulate cortex |  |  |  |
|  | Left | Cuneus, Calcarine fissure and surrounding cortex | -2, -92, 18 | 4.33 | 469 |
|  | Right | Cuneus |  |  |  |
